# Induction of mastitis by cow-to-mouse fecal and milk microbiota transplantation causes microbiome dysbiosis and genomic functional perturbation in mice

**DOI:** 10.1186/s42523-022-00193-w

**Published:** 2022-07-06

**Authors:** M. Nazmul Hoque, M. Shaminur Rahman, Tofazzal Islam, Munawar Sultana, Keith A. Crandall, M. Anwar Hossain

**Affiliations:** 1 Department of Gynecology, Obstetrics and Reproductive Health, Bangabandhu Sheikh Mujibur Rahman Agricultural University (BSMRAU), Gazipur, 1706 Bangladesh; 2 Department of Microbiology, Jashore University of Science and Technology, Jashore, 7408 Bangladesh; 3 Institute of Biotechnology and Genetic Engineering (IBGE), BSMRAU, Gazipur, 1706 Bangladesh; 4 Department of Microbiology, University of Dhaka, Dhaka, 1000 Bangladesh; 5 Computational Biology Institute and Department of Biostatistics and Bioinformatics, Milken Institute School of Public Health, The George Washington University, Washington, DC, 20052 USA; 6 Jashore University of Science and Technology, Jashore, 7408 Bangladesh

**Keywords:** Cows, Mice, Mastitis, Feces, Milk, Mammary gland, Microbiomes, Transplantations, Dysbiosis

## Abstract

**Background:**

Mastitis pathogenesis involves a wide range of opportunistic and apparently resident microorganims including bacteria, viruses and archaea. In dairy animals, microbes reside in the host, interact with environment and evade the host immune system, providing a potential for host-tropism to favor mastitis pathogenesis. To understand the host-tropism phenomena of bovine-tropic mastitis microbiomes, we developed a cow-to-mouse mastitis model.

**Methods:**

A cow-to-mouse mastitis model was established by fecal microbiota transplantation (FMT) and milk microbiota transplantation (MMT) to pregnant mice to assess microbiome dysbiosis and genomic functional perturbations through shotgun whole metagenome sequencing (WMS) along with histopathological changes in mice mammary gland and colon tissues.

**Results:**

The cow-to-mouse FMT and MMT from clinical mastitis (CM) cows induced mastitis syndromes in mice as evidenced by histopathological changes in mammary gland and colon tissues. The WMS of 24 samples including six milk (CM = 3, healthy; H = 3), six fecal (CM = 4, H = 2) samples from cows, and six fecal (CM = 4, H = 2) and six mammary tissue (CM = 3, H = 3) samples from mice generating 517.14 million reads (average: 21.55 million reads/sample) mapped to 2191 bacterial, 94 viral and 54 archaeal genomes. The Kruskal–Wallis test revealed significant differences (*p* = 0.009) in diversity, composition, and relative abundances in microbiomes between CM- and H-metagenomes. These differences in microbiome composition were mostly represented by *Pseudomonas aeruginosa*, *Lactobacillus crispatus*, *Klebsiella oxytoca*, *Enterococcus faecalis*, *Pantoea dispersa* in CM-cows (feces and milk), and *Muribaculum* spp., *Duncaniella* spp., *Muribaculum intestinale*, *Bifidobacterium animalis*, *Escherichia coli, Staphylococcus aureus*, *Massilia oculi*, *Ralstonia pickettii* in CM-mice (feces and mammary tissues). Different species of *Clostridia*, *Bacteroida*, *Actinobacteria*, *Flavobacteriia* and *Betaproteobacteria* had a strong co-occurrence and positive correlation as the indicator species of murine mastitis. However, both CM cows and mice shared few mastitis-associated microbial taxa (1.14%) and functional pathways regardless of conservation of mastitis syndromes, indicating the higher discrepancy in mastitis-associated microbiomes among lactating mammals.

**Conclusions:**

We successfully induced mastitis by FMT and MMT that resulted in microbiome dysbiosis and genomic functional perturbations in mice. This study induced mastitis in a mouse model through FMT and MMT, which might be useful for further studies- focused on pathogen(s) involved in mastitis, their cross-talk among themselves and the host.

**Graphical Abstract:**

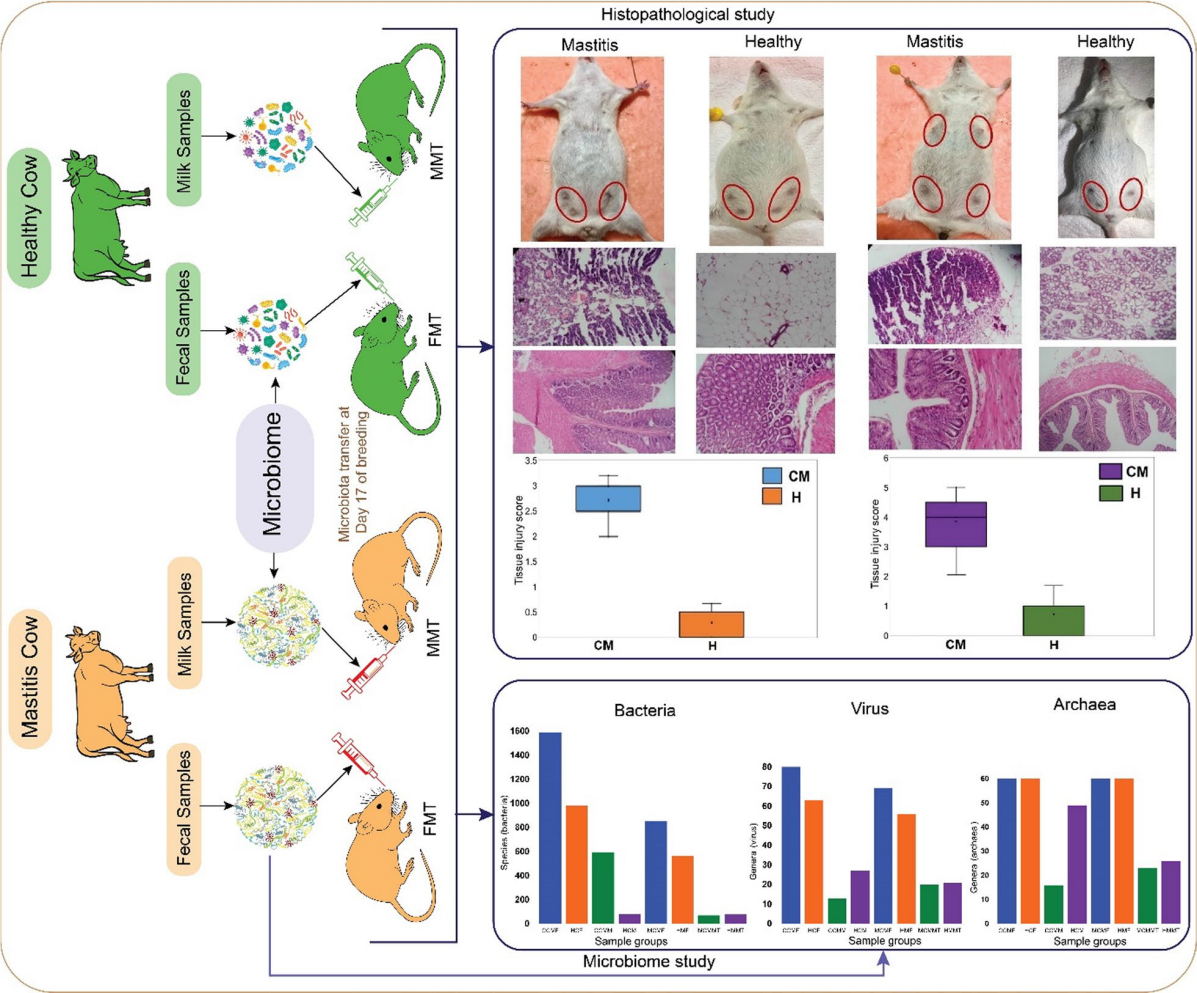

**Supplementary Information:**

The online version contains supplementary material available at 10.1186/s42523-022-00193-w.

## Introduction

Mastitis is one of the most prevalent infectious diseases in the dairy animals worldwide. It has a negative impact on the agro-economy due to reduced milk production, early culling, and high therapeutic costs [1–4]. The disease is caused by a wide range of apparently resident and opportunistic microbes including bacteria, viruses, and archaea of variable origin, where the severity and outcome of the disease depends on the cross-talk between host and pathogen [5–8]. During mastitis, an imbalance of microbiomes occurs due to the inclusion of the pathogenic and opportunistic microorganisms in susceptible mammary glands. This invading encroachment is favoured by the compromised immune status of the host and the interactions between the opportunistic pathogens and the resident microbiota of the mammary gland [5, 7–11]. Depending on the host–pathogen interactions [12–14], bovine mastitis can manifest clinical features of apparent changes in the color of milk, swelling, redness, warmth and pain in the affected udder, systemic symptoms like fever and anorexia and sometimes death due to toxemia [15, 16]. Despite intensive research and implementation of various strategies over the last few decades to manage mastitis, the control of this dairy disease is still elusive because of the dynamic changes in the etiology [3]. Moreover, microbial communities colonizing the mammary gland have evolved several novel mechanisms to facilitate their opportunistic proliferation manifesting the pathogenesis of CM [5, 7, 9]. The microbiomes of bovine CM comprise both contagious udder pathogens including *Staphylococcus aureus*, *Streptococcus agalactiae*, *Streptococcus dysgalactiae*, *Mycoplasma* spp., and *Corynebacterium bovis* [7, 11], and environmental pathogens such as *Escherichia coli*, *Klebsiella pneumoniae*, *Klebsiella oxytoca*, *Enterobacter aerogenes*, *Streptococcus dysgalactiae*, and *Streptococcus uberis* [8, 17]. However, microbiomes of the mammary glands and milk are considered to be highly similar, and the origin of microbes in the milk could be deep inside the upper parts of the mammary gland. Therefore, it is very likely that many of these microbes can migrate to the mammary gland from extra-mammary sites such as the gut, via the entero-mammary axis and/or the environment [17, 18].

In the last decade, the advent of shotgun metagenomics and downstream bioinformatics analyses allow for robust surveys of host-microbiome interactions in dairy populations [5, 7, 10]. A large body of literature suggests that disruption of gut microbiota homeostasis is linked to metabolic, immune, gastrointestinal, and mammary gland diseases [5, 19–22]. Previous studies demonstrated that changes in the gut microbiota can result in proliferation of specific pathogenic bacteria which can thereafter enter the mammary gland through the entero-mammary pathway [23–25]. The colonization of bovine gut microbiota into germ free (GF) mouse model demonstrates the influence of bovine gut microbes on the development of mastitis [5, 24, 25]. The usefulness of a mouse mastitis model as a complementary tool to investigate differences between bovine associated coagulase-negative *Staphylococci* (CNS) has already been established [26]. Ma et al. [5] recently reported that cow-to-mouse fecal microbiota transplantation can modulate intestinal microbiome dysbiosis, which was one cause of mastitis. In this study, we found that in addition to fecal microbiota, CM milk microbiota transplantation is one of the important cuases of mastitis. Therefore, the cows-to-mouse mastitis milk microbiota transplantations (FMT and MMT) might be useful for further molecular biological studies on mastitis using the mouse model. Previous studies reported that humanized gnotobiotic mouse models have contributed to advancements in biomedicine, bridging the gap between human and animal gut pathophysiology [25, 27, 28].

In the pathophysiology of bovine mastitis, dysbiosis of the milk microbiomes occurs with an increase in opportunistic pathogens, and a reduction in healthy milk commensal microbes [8, 29]. The opportunistic pathogens of the gut use very efficient strategies to evade host defenses in order to colonize and invade mammary tissues. Thereafter, damage occurs to mammary epithelial cells and disruption of the cow immune system causes clinical episodes of CM [7, 30]. Therefore, to investigate the host-tropism of bovine mastitis microbiomes using mouse model, this study attempted to explore whether dysbiosis of fecal and milk microbiota can lead to clinical episodes of mastitis in pregnant mice. In the present study, we induced mastitis in mice by transplantation of bovine fecal or milk microbiota from CM cows into GF pregnant mice and investigated the changes in the fecal and mammary tissue microbiota using a high-throughput WMS followed by metagenomic and metabolic functional analyses along with histopathological changes in mice mammary gland and colon tissues (Graphical abstract). Our study revealed that both CM cows and mastitis induced mice shared a limited number of microbial taxa and related genomic functional potentials. We also observed a high degree of discrepancy in the microbiomes in milk, mammary tissue and feces of the mastitis affected animals. The developed mouse model would be useful for identification of the primary pathogen(s) of bovine mastitis, and their molecular interactions with hosts that are critical for sustainable management of this economically important disease in the dairy industry worldwide. Although, the baseline data presented here are promising, further studies are recommended using a larger sample size, and with the inclusion of gut/rumen microbiome sampling in addition to the milk samples for direct testing of microbial transfer across entero-mammary axis to confirm the dysbiosis of microbiomes and associated genomic functional features.

## Results

### Fecal or milk microbiome transplantion from mastitis cows induced mastitis in GF mice

**Fig. 1:**
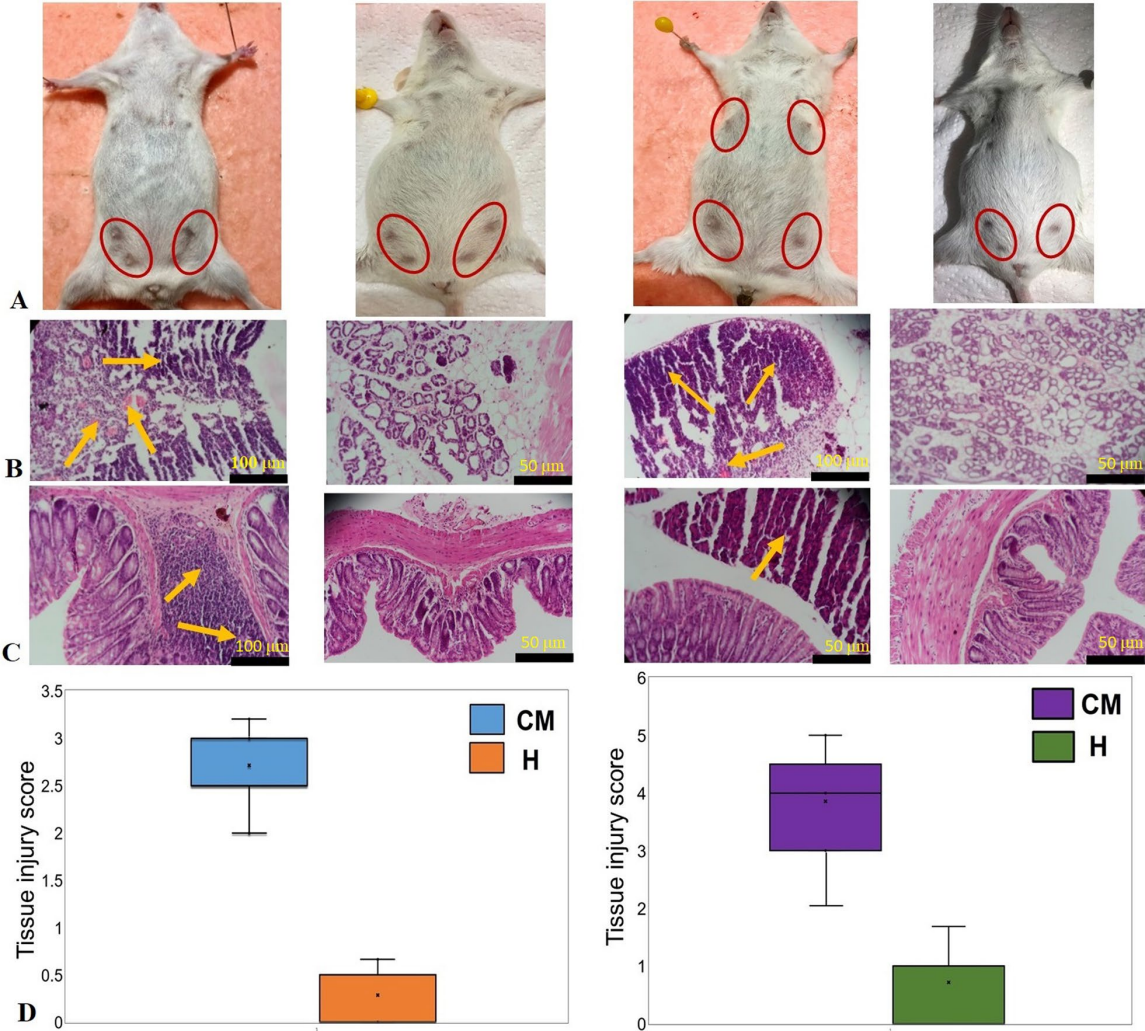
Histopathological analysis of mouse gut and mammary tissues after microbiota transplantation. **A** Pathological changes in mammary gland surface, where different abdominal mammary glands were found as swollen and red in the mastitis (CM) group of mice on Day 27 after FMT (fecal microbiota transplantion) and MMT (milk microbiota transplantion). Mammary glands of mouse are highlighted by red circles. **B** Representative photomicrographs of mammary epithelial tissue after haematoxylin and eosin staining (×100 magnification). The lesion in the mammary epithelial cells is characterised by a central area of necrosis, broken lobules, damaged acinuses and destroyed epithelial cells, with with large numbers of inflammatory cells, predominantly neutrophils (dark bluish) and macrophages (red) (yellow arrows), in the mammary lobule, supporting connective tissue and linning of the epithelium. **C** Representative photomicrographs of colon (crypts, lamina propria, muscularis mucosae and submucosa) tissue after haematoxylin and eosin staining (×100 magnification). Histopatholaogical changes in the colon tissue include moderate to severe inflammatory cell infiltration into mucosa and submucosa, disorder in mucosal structure: epithelial necrosis, extension of the subepithelial space, and structural damage of villi (yellow arrows). Scale bars: 100 μm and 50 μm. **D** The tissue injury scores in the mammary gland and colon. The X-axis represents the groups of samples (CM and H) while the Y-axis denotes the injury scores as measured through Chiu Scoring System (×100 magnification)

To elucidate whether CM-associated cow microbiota could develop mastitis syndrome in mice, fecal or milk microbiota from seven CM and five H cows were transplanted to 40 GF pregnant mice. The sample groups were- CCMF: cow clinical mastitis feces; HCF: healthy cow feces; CCMM: cow clinical mastitis milk; HCM: healthy cow milk; MCMF: mouse clinical mastitis feces; HMF: healthy mouse feces; MCMMT: mouse clinical mastitis mammary tissue; and HMMT: healthy mouse mammary tissue (Additional file 1). Among the 40 recipient mice, 20 underwent to FMT and MMT from CM samples while the remaining 20 were treated with H cow fecal and milk microbiota (controls). By comparing the post-intervention (FMT and MMT) inflammatory responses among the four groups, we found that fecal microbiota from CM cows induced a higher incidence of mastitis (90.0%) in mice compared to milk microbiota (80.0%). However, clinical signs of CM were not apparent before 10 days-post challenges (i.e., before 27 days of gestation). Remarkably, none of the mice in the control groups (either H-cows FMT or MMT) developed mastitis syndromes. Comparison of murine post-microbiota transplantation inflammatory responses revealed that gut and milk microbiota from CM cows induced a much greater inflammatory (of mammary gland and colon) response than those from healthy cows. On mammary gland surface, severe gross pathological changes (e.g., swelling and congestion of mammary glands) that corresponded to CM were observed in the mastits groups (CCMF and CCMM), however no pathological changes were visually apparent in healthy groups i.e., HCF or HCM (Fig. 1A). The gross syndrome of CM was further supported and confirmed by histopathological changes in the mammary and colon tissues. The CM-associated histopathological changes included damage of the mammary gland tissue (e.g., loss of architecture of alveoli, haemorrhages, involuted alveoli, degenerative changes in the epithelium of the alveoli and ducts, mammary alveolus thickening, hyperemia, and edema) and extent of inflammatory cell infiltration (i.e., infiltration of polymorphonuclear cells). For example, observable histopathological lesions under hematoxylin–eosin staining includes broken lobules of the mammary gland, damaged acinuses, and destroyed epithelial cells, with inflammatory cells including macrophages, neutrophils, and blood cells detected in the mammary lobule, supporting connective tissue and linning of the epithelium (Fig. 1B); on the other hand, in control groups (HCF or HCM), no pathological changes were apparent. In addition, predominant changes in the colon tissues of the CM mice (Fig. 1C) included moderate to severe inflammatory cell infiltration into mucosa and submucosa, disorder in mucosal structure (epithelial necrosis, extension of the subepithelial space, and structural damage of villi). Conversely, the mice receiving H cows fecal and milk microbiota did not show such pathological changes in the colon tissues and exhibited normal intestinal mucosa with well-arranged villi structure (Fig. 1C). Moreover, the CM mice had significantly higher (*p* = 0.0013, Kruskal–Wallis test) pathological grade of injury in their mammary gland and colon tissues than those of H mice (Fig. 1D). Generally, healthy mammary tissue possesses more fat vacuoles than mastitis udder [31], and thus, occurrence of mastitis was not affected by the lactation periods.

### Microbiome structure and composition in gut, milk and mammary tissues differed in cows and mice

**Fig. 2:**
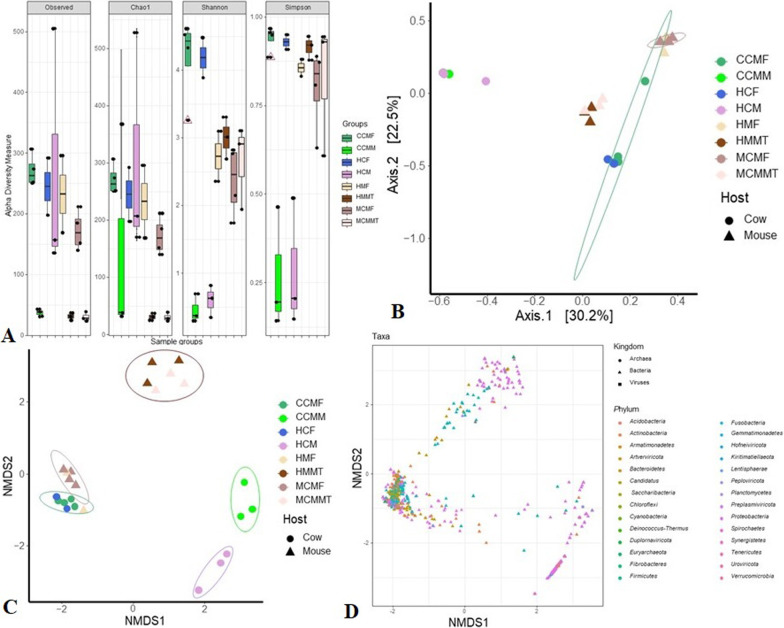
Microbiome diversity and composition in CM and H samples of cows and mice after FMT (fecal microbiota transplantion) and MMT (milk microbiota transplantion). **A** Alpha diversity measures with Observed, Chao1, Shannon and Simpson indices to visualize the difference in microbiota structure in fecal, milk and mammary tissues of cows and mice. **B** Principal coordinate analysis (PCoA) plot (measured on the Bray–Curtis distance method) showing significant microbiome diversity in different hosts i.e., cows and mice (*p* = 0.005, Kruskal–Wallis test) and sample groups (*p* = 0.001, Kruskal–Wallis test). **C** Non-metric multi-dimensional scaling (NMDS) plot (measured with Weighted-UniFrac method) representing significant microbial beta diversity in different hosts (cows and mice) (*p* = 0.005, Kruskal–Wallis test) and sample groups (*p* = 0.001, Kruskal–Wallis test). **D** NMDS plot showing phylum level microbiome (archaea, bacteria and viruses) diversity in all samples of the study metagenomes. The sample groups are- CCMF: cows clinical mastitis feces; HCF: healthy cows feces; CCMM: cows clinical mastitis milk; HCM: healthy cows milk; MCMF: mouse clinical mastitis feces; HMF: healthy mouse feces; MCMMT: mouse clinical mastitis mammary tissue; and HMMT: healthy mouse mammary tissue

To test for differences in taxonomic diversity and structure of microbiomes, we analyzed the WMS data using an open-source cloud-based metagenomic mapping-based method of IDSeq [32]. We found significant differences in microbial α-diversity (i.e., within-sample diversity) estimated through observed species in each sample (i.e., richness), Chao1, Shannon, and Simpson indices in the CM and H samples regardless of the host. The species level α-diversity remained significantly higher in H control samples of both cows (*p* = 0.0079, Kruskal–Wallis test) and mice (*p* = 0.0068, Kruskal–Wallis test) compared to CM samples (Fig. 2A). The PCoA plot based on Bray–Curtis distances (Fig. 2B) and NMDS plot based on weighted UniFrac distances (Fig. 2C), showed significant microbial disparity between CM and H samples (*p* = 0.001, Kruskal–Wallis test) and hosts (i.e., cows and mice) (*p* = 0.005, Kruskal–Wallis test). The phylum-level microbiome composition also showed distinct differences (*p* = 0.002, Kruskal–Wallis test) across the detected microbial domains (i.e., bacteria, archaea and viruses) (Fig. 2D).

**Fig. 3:**
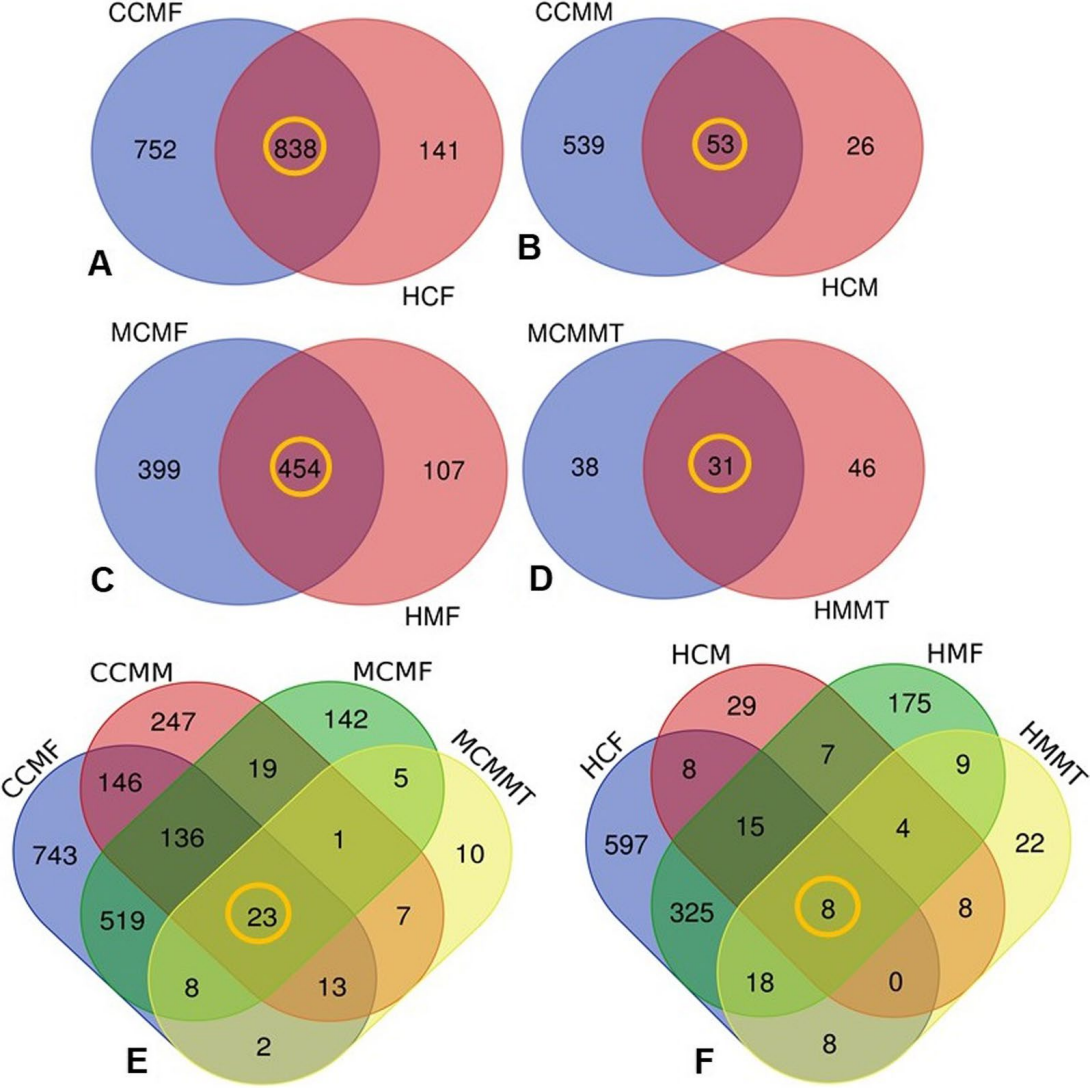
Taxonomic composition of bacteriomes. Venn diagrams representing the core unique and shared microbiomes in cows faecal material and milk samples, and mouse faecal material and mammary tissues. Bacterial species detected in **A** cows faecal material, **B** cows milk, **C** mouse faecal material and **D** mouse mammary tissues. **E** Unique and shared bacterial species in CM, and **F** unique and shared bacterial species in H metagenomes of both cows and mice. Bacterial species shared in cow and mouse mastitis (CCMM, CCMF, MCMF, MCMMT) and healthy (HCM, HCF, HMF, HMMT) metagenomes are highlighted in yellow color circles. More information on the taxonomic result is also available in Additional file 2

At the domain level, bacteria were the most abundant (99.78%) microbial community followed by viruses (0.13%) and archaea (0.09%) (Additional file 2). In this study, we detected 1731 bacterial species including 1590 and 979 in CM and H cow fecal samples, respectively (Fig. 3A), of which 43.44% species had sole association with CM (Fig. 3A, Additional file 3). Similarly, 618 species of bacteria were identified in cows milk including 592 and 79 species in CM and H milk samples, respectively, and the CM samples had sole association of 87.22% species (Fig. 3B, Additional file 3). Conversely, 1065 species including 853 in CM and 721 in H fecal samples of mice were detected (Fig. 3C), and of them, the CM fecal samples had sole association of 32.30% species (Fig. 3C). Likewise, 115 species of bacteria were identified in mouse mammary tissue including 69 in CM and 77 in H tissues (Fig. 3D), and the CM mice mammary tissues had sole association of 33.04% species (Fig. 3D, Additional file 3). Comparing the bacterial taxa in CM and H sample in both hosts, we found that only 1.14% and 0.65% bacterial species shared across the CM (Fig. 3E) and H (Fig. 3F) samples, respectively.

In addition to bacterial fraction of the microbiomes, we detected 94 viral (Additional file 4), and 54 archaeal (Additional file 5) genera from both cows and mice (Additional file 3). By comparing these genera across the sample categories, we found 78 and 62 genera in cow CM and H samples, respectively, and majority of these genera (75.0%) were found to be shared between CM and H samples. The milk samples of both CM and H cows harboured 29 viral genera (CM = 13 and H = 26) and, only 10.44% genera had sole association with CM. Likewise, 74 viral genera were detected in mice fecal samples (CM = 69, H = 57), of which 70.27% genera were shared between the conditions. The mammary tissue of mice possessed only 24 viral genera (CM = 20, H = 21), and among these genera, 70.84% were shared between the conditions. In this study, we found that fecal samples from both cows and mice harboured 54 archaeal genera (CM = 54, H = 54, in each category), and of them, 100% of the genera were found to be shared between the conditions (CM and H) (Additional file 5). Moreover, 32 and 46 archaeal genera were detected in cow’s milk and mice mammary tissues, respectively. In this study, the facal and milk samples of both CM and H cows were found to share 17 archaeal genera (Additional file 5) while the facal and mammary tissue samples of both CM and H mice shared 16 archaeal genera (Additional file 5).

### Gut and milk bacteria of mastitis cows are distinct from those of healthy cows

To test associations between microbiomes (gut and milk) and clinical mastitis (CM), fecal and milk microbiota from seven crossbred Holstein cows diagnosed with CM were compared to five physically similar, age-matched, crossbred Holstein cows that served as the control (Additional file 1; Materials and method). We found significant differences (*p* = 0.009, Kruskal–Wallis test) in the relative abundance of the bacterial species in fecal and milk samples of dairy cows. Among the detected bacterial species, *Pseudomonas aeruginosa* (21.0%), *Lactobacillus crispatus* (16.10%), *Klebsiella oxytoca* (10.34%), *Enterococcus faecalis* (10.0%), *Nocardia pseudobrasiliensis* (5.0%), *Lactobacillus vaginalis* (4.85%), *Clostridioides difficile* (4.23%), *Ralstonia insidiosa* (4.0%), *Bifidobacterium pseudolongum* (3.80%), *Muribaculum* sp. (2.35%), *Duncaniella* sp. (2.32%), and *Duncaniella dubosii* (2.0%) were the top abundant species in the CM-fecal samples of cows (Fig. 3, Additional file 6). Conversely, *P. aeruginosa* (32.7%), *E. faecalis* (30.05%), *Lachnospiraceae bacterium* (3.03%), *Clostridiales bacterium* (2.28%), and *Phocaeicola dorei* (2.15%) had higher relative abundances in H-cow fecal samples compared to those of CM cows (Fig. 3, Additional file 6). Similarly, the CM-cow milk samples were dominated by *Pantoea dispersa* (24.29%), *K. oxytoca* (20.68%), *Actinoalloteichus* sp. (11.63%), *N. pseudobrasiliensis* (8.87%), *Staphylococcus aureus* (4.7%), *C. botulinum* (4.19%), *Acinetobacter baumannii* (4.0%), *Acinetobacter johnsonii* (3.5%), *K. pneumoniae* (3.44%) and *Escherichia coli* (3.02%). On the contrary, *P. aeruginosa* had the highest relative abundance (40.35%) in H-cow milk samples followed by *P. dispersa* (13.86%), *A. baumannii* (11.0%), *Prevotella melaninogenica* (10.0%), *Actinoalloteichus* sp. (5.25%), *K. oxytoca* (4.0%) and *N. pseudobrasiliensis* (3.05%). The remaining bacterial species detected from the metagenomic data had relatively lower (< 3.0%) abundances and remained mostly abundant in CM-associated fecal and milk samples of cows (Fig. 3, Additional file 6).

### Cow-to-mouse FMT and MMT reveal distinct gut and mammary gland bacteria between mastitis and healthy mice

**Fig. 4:**
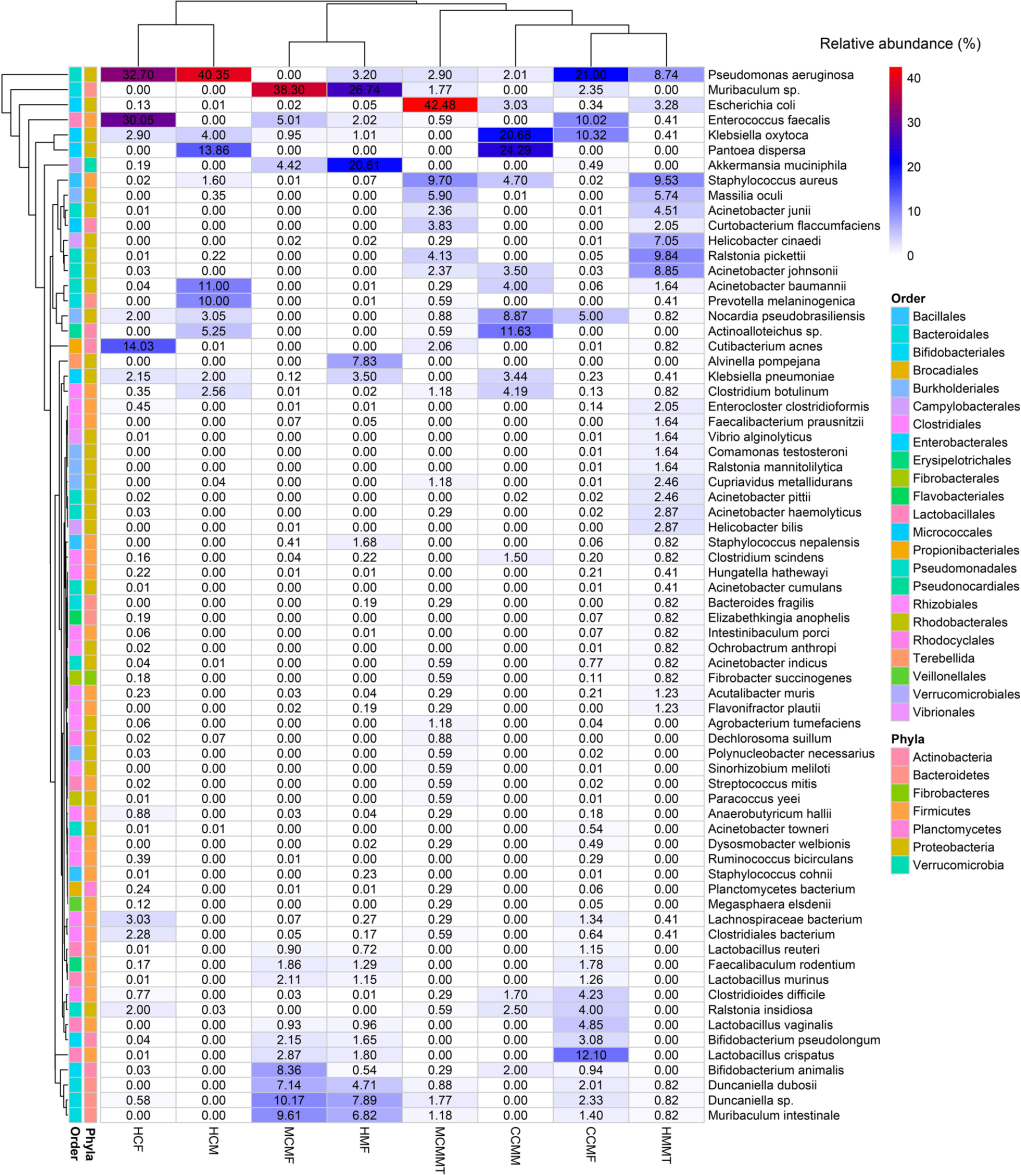
Species-level taxonomic clustering of bacteria. The heatmap shows the hierarchical clustering of sample groups based on the relative abundance of the top ranked 70 bacterial species identified in cow and mouse mastitis (CCMM, CCMF, MCMF, MCMMT) and healthy (HCM, HCF, HMF, HMMT) metagenomes. The relative values in the heatmap (after normalization), depicted by colors, indicate the aggregation degree or content of bacterial species among samples at the phylum and order level. The color bar (light blue to red) displays the row Z-scores (0–40): red color indicates high abundance, light blue color low abundance. The color of the squares on the left indicates the relative abundance of the bacterial species in each group. The distribution and relative abundance of the bacterial species in the study metagenomes are also available in Additional file 2

We further investigated whether cow-to-mouse FMT and MMT treatment could produce distinct disease outcomes among the challenged mice. The WMS of both fecal and mammary tissues obtained from seven-CM and five-H mice at Day 27 of gestation (Methods) showed distinct changes in both composition and relative abundances of bacteria at the species level. The CM-mice fecal samples had a higher number of bacterial species (n = 853) than H-mice fecal samples (n = 561). The CM-related fecal metagenome of mice (MCMF) was dominated by *Muribaculum* sp. (38.30%), *Duncaniella* sp. (10.17%), *Muribaculum intestinale* (9.61%), *Bifidobacterium animalis* (8.36%), *D. dubosii* (7.14%), *E. faecalis* (5.0%), *Akkermansia muciniphila* (4.42%), *L. crispatus* (2.87%), *B. pseudolongum* (2.15%) and *Lactobacillus murinus* (2.10%) (Fig. 3, Additional file 6). In contrast, the H-mice fecal metagenome (HMF) was dominated by *Muribaculum* sp. (26.74%), *A. muciniphila* (20.61%), *Duncaniella* sp. (7.89%), *M. intestinale* (6.82%), *D. dubosii* (4.71%), *K. pneumoniae* (3.5%), *P. aeruginosa* (3.2%), and *E. faecalis* (2.0%), and rest of the species detected in both groups had comparatively lower relative abundances than that of CM-mice (Fig. 4, Additional file 6). The mammary tissues of the challenged mice had a significantly lower number of bacterial species compared to fecal samples (115 vs. 1065). In the mammary tissue of CM mice (MCMMT), *E. coli* (42.48%) was identified as the single most prevalent species followed by *S. aureus* (9.7%), *Massilia oculi* (5.90%), *Ralstonia pickettii* (4.13%), *Curtobacterium flaccumfaciens* (3.84%), *P. aeruginosa* (2.9%), *A. johnsonii* (2.37%), *A. junii* (2.36%), and *Cutibacterium acnes* (2.06%) (Fig. 4). Conversely, the H mice mammary tissue metagenome (HMMT) was mostly dominated by *Ralstonia pickettii* (9.84%), *S. aureus* (9.53%), *A. johnsonii* (8.85%), *P. aeruginosa* (8.74%), *Helicobacter cinaedi* (7.049%), *M. oculi* (5.74%), *A. junii* (4.51%), and *E. coli* (3.28%). The remaining species detected in both MCMMT and HMMT metagenomes had relatively lower (< 3.0%) abundances (Fig. 4, Additional file 6).

### Indicator and shared bacterial taxa in the mastitis and healthy mice metagenomes

**Fig. 5:**
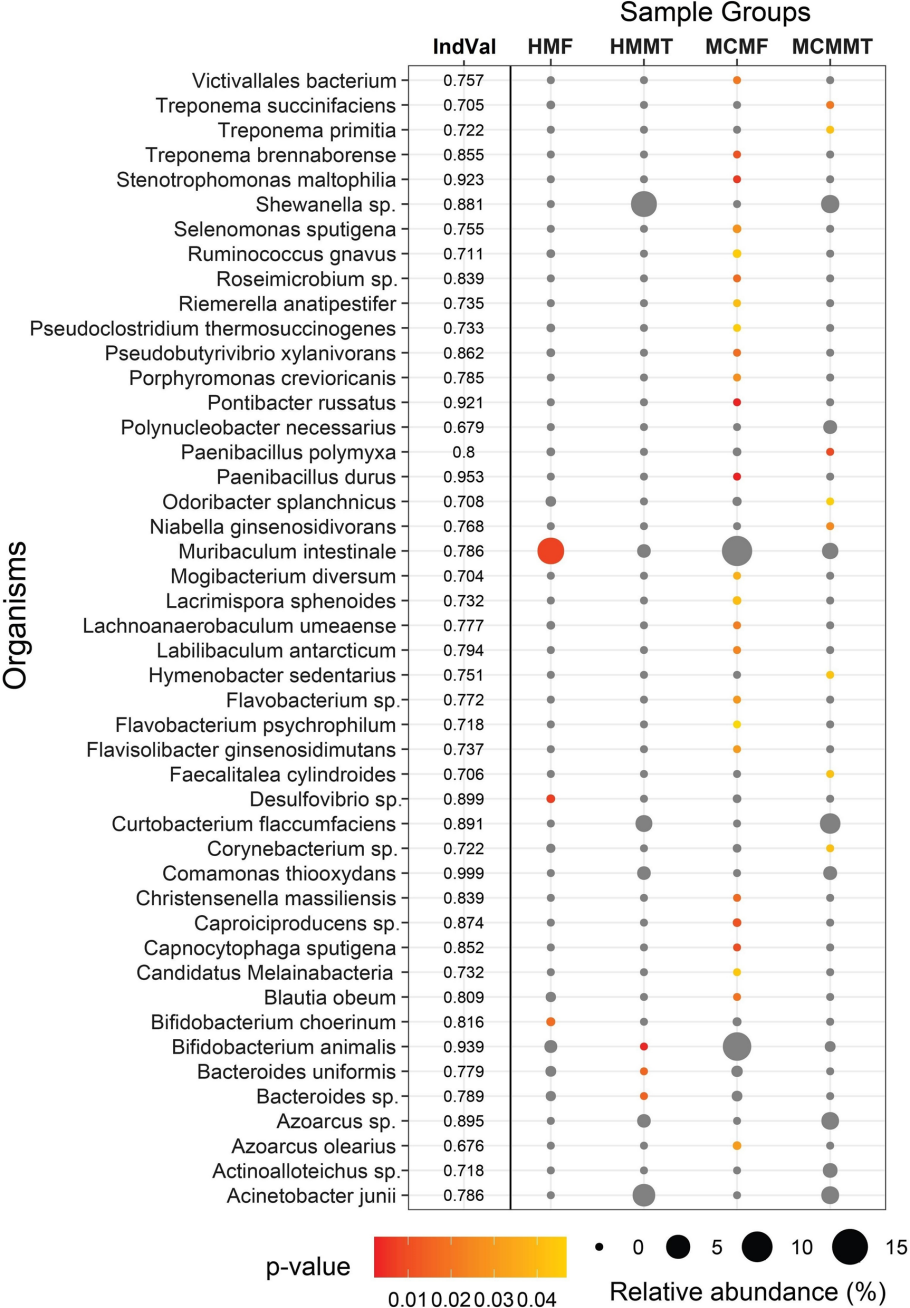
Indicator species in both mastitis (MCMF and MCMMT) and healthy (HMF and HMMT) mice metagenomes. Differentially enriched indicator species in different sample groups of mouse mastitis model. Indicator values are shown next to the taxonomic information for the indicator taxa as indicated by Indicator. Only highly significant indicator values (IndVal > 0.6, *p* < 0.01) are displayed. Size of bubble is proportional to the mean relative abundance of each species in the corresponding sample group, and the color scale bar shows indicator value for each taxon. Red color indicate the level of significance for which group the taxon is an indicator. Grey symbols indicate group that contain a taxon, but for which that taxon is not an indicator taxa

The indicator species analysis identified 46 differentially abundant (IndVal values ≥ 0.6, *p* < 0.01) bacterial species in mouse CM (MCMF and MCMMT) and H (HMF and HMMT) metagenomes (Fig. 5). In this study, mice CM samples showed the highest number of differentially enriched species (including 24 species in MCMF samples and eight species in MCMMT samples). Among the indicator species identified, *Paenibacillus durus*, *Stenotrophomonas maltophilia*, *Pontibacter russatus*, *Caproiciproducens* sp. NJN-50, *Pseudobutyrivibrio xylanivorans*, *Treponema brennaborense*, *Capnocytophaga sputigena*, *Roseimicrobium* sp. ORNL1, *Christensenella massiliensis*, and *Blautia obeum* were the top scoring (IndVal ≥ 0.8, *p* = 0.002) species in MCMF samples. Similarly, *P. polymyxa*, *Niabella ginsenosidivorans*, *Hymenobacter sedentarius* and several species that were classified at higher taxonomic ranking (IndVal ≥ 0.7, *p* = 0.03) were differentially abundant in the MCMMT samples. Remarkably, indicator species analysis confirmed that *P. durus*, *S. maltophilia*, *P. russatus* and *Paenibacillus polymyxa* were all good indicators of the murine CM (Fig. 5, Additional file 2). On the contrary, the H-mice samples had only seven differentially enriched bacterial species. The most abundant species (IndVal ≥ 0.78, *p* = 0.01) in the HMF samples were *Bifidobacterium choerinum*, *Desulfovibrio* sp. IOR2, and *M. intestinale* while *Azoarcus olearius*, *Bacteroides* sp. A1C1, *B. uniformis* and *B. animalis* were enriched (IndVal ≥ 0.79, *p* = 0.01) in HMMT samples (Fig. 5).

**Fig. 6:**
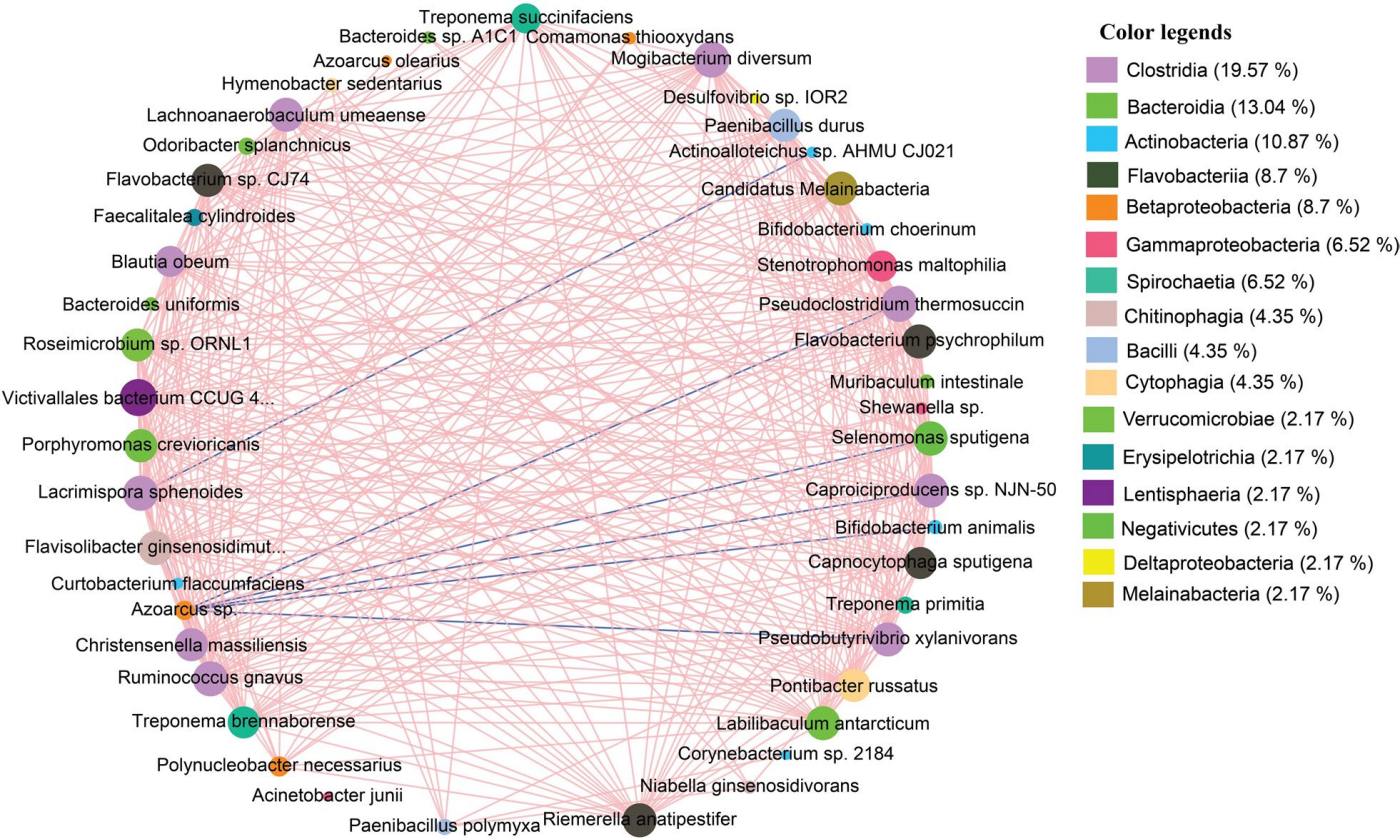
Microbiome co-occurrence in both mastitis (MCMF and MCMMT) and healthy (HMF and HMMT) mice metagenomes. Network of co-occurring patterns among dominant species based on the Spearman’s (r > 0.5 and *p* < 0.01) correlation tests. Nodes represented dominant species and identified by different colors. The size of each node is proportional to the number of connections, and the thickness of each connection between two nodes (edge) is proportional to the value of Spearman’s correlation coefficients. The nodes are colored by phylum, and labels according to the taxonomic affiliations. The positive correlation (97.74%) is represented by the red lines while the blue lines represent negative correlation (2.26%). The more connections, the more association is associated with other members of the community

A microbial co-occurrence network was built based on correlations of relative abundance of the indicator species between mice-CM and -H samples. The network analysis (Fig. 6) presented 46 nodes and 1449 edges (significant positive correlations, r > 0.6 and Spearman’s corrected *p* = 0.001), all connected into one cluster with a clustering coefficient of 0.503. In the co-occurrence network, *Clostridia* had the highest number of edges (19.57%) followed by *Bacteroida* (13.04%), *Actinobacteria* (10.87%), *Flavobacteriia* (8.7%), *Betaproteobacteria* (8.7%), *Gammaproteobacteria* (6.52%), and *Spirochaetia* (6.52%), indicating strong co-occurrence among the species of these classes (Fig. 6). *Pseudoclostridium thermosuccinogenes* and *Pseudobutyrivibrio xylanivorans* (phylum: *Firmicutes*, class: *Clostridia*) had the highest co-occurrence (Correlation coefficient: 0.55, *p* = 0.005, in each) showing positive correlation with 45 indicator species and had only a negative correlation with *Azoarcus* species. Likewise, *Labilibaculum antarcticum* (phylum: *Bacteroidetes*, class: *Bacteroida*) was correlated with 25 bacterial species including *P. russatus*, *Polynucleobacter necessaries*, *Treponema succinifaciens* etc. and had no negative correlation with other indicator species (Fig. 6). In contrast, *Azoarcus* sp. and *Lacrimispora sphenoides* were mutually exclusive in the mouse habitats, and their abundance in mouse gut and mammary tissues was negatively correlated (Fig. 6).

### CM-associated changes in viral and archaeal fraction (genus-level) of microbiomes in mice

**Fig. 7:**
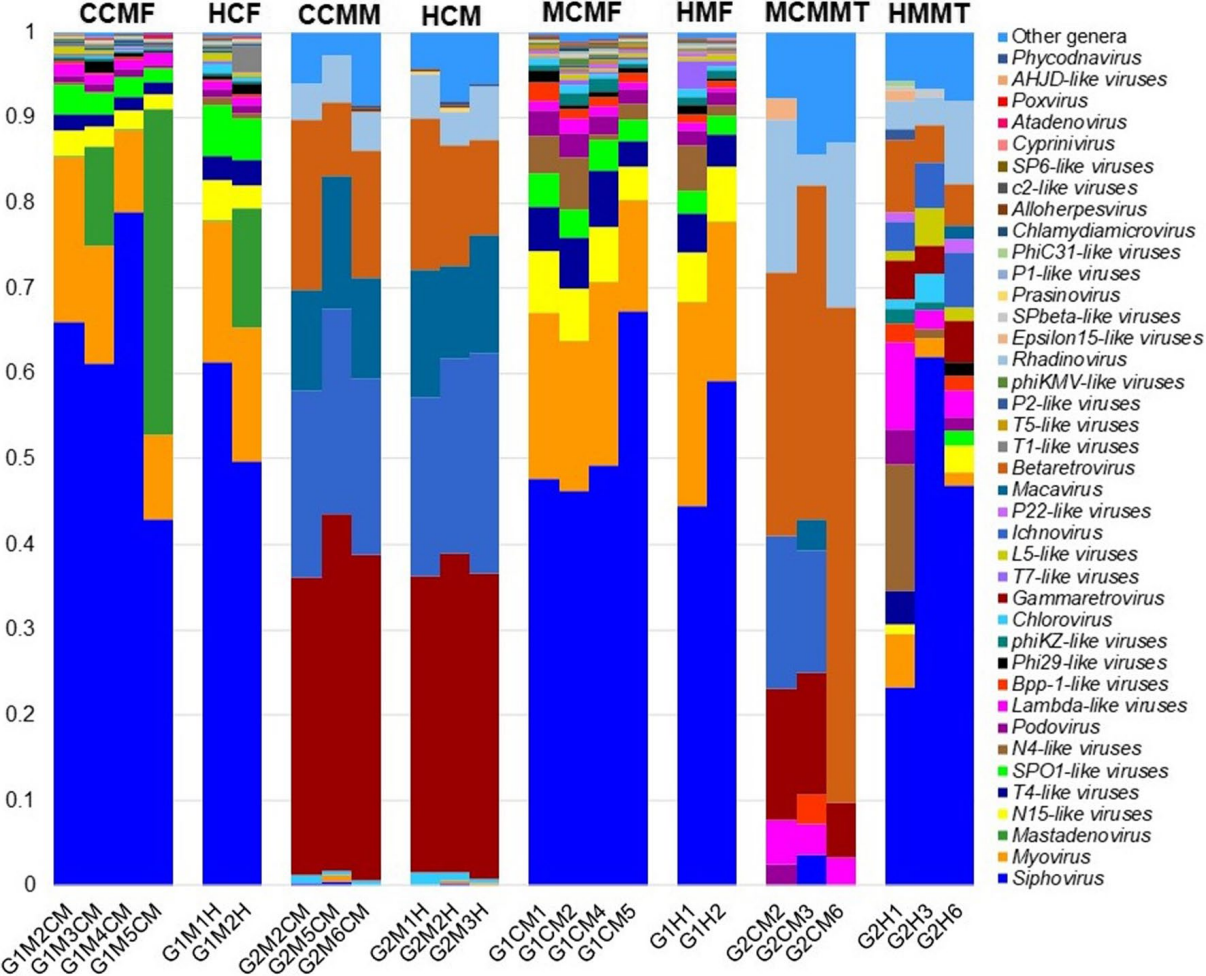
The genus-level taxonomic profile of viruses identified in cow and mouse mastitis (CCMF, CCMM, MCMF, MCMMT) and healthy (HCF, HCM, HMF, HMMT) samples. Stacked bar plots showing the relative abundance and distribution of the 40 top abundant viral genera, with ranks ordered from bottom to top by their decreasing proportion of relative abundances, with the remaining genera keeping as ‘Other genera’. Each stacked bar plot represents the abundance of viral genera in each sample of the corresponding category. Notable differences in viral populations are those where the taxon is abundant in clinical mastitis (cow and mouse) samples, and effectively undetected in the healthy (cow and mouse) controls. The distribution and relative abundance of the viral genera in the study metagenomes are also available in Additional file 2

**Fig. 8:**
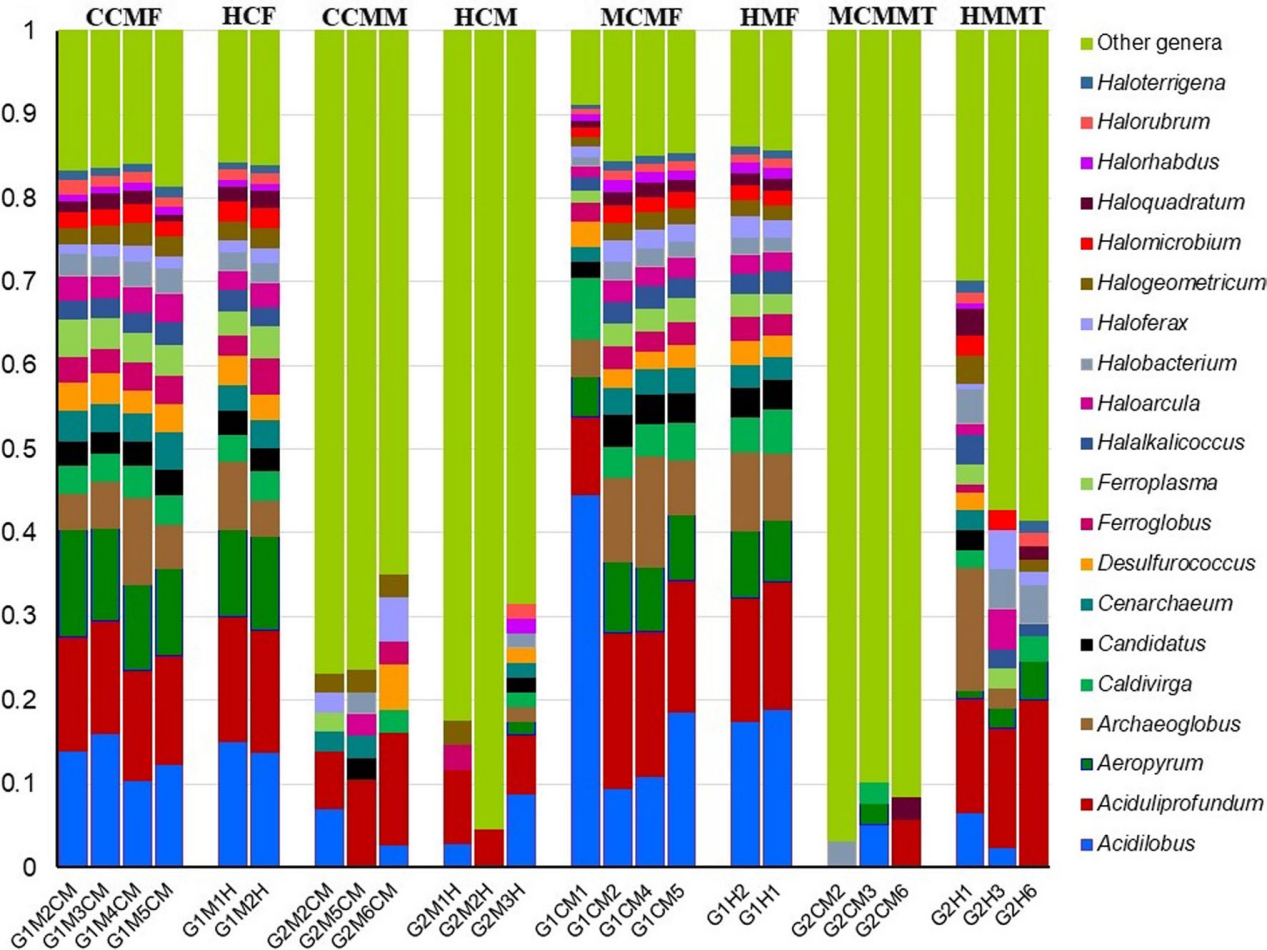
The genus-level taxonomic profile of archaea identified in cow and mouse mastitis (CCMF, CCMM, MCMF, MCMMT) and healthy (HCF, HCM, HMF, HMMT) samples. Stacked bar plots showing the relative abundance and distribution of the 20 top abundant archaeal genera, with ranks ordered from bottom to top by their decreasing proportion of relative abundances, with the remaining genera keeping as ‘Other genera’. Each stacked bar plot represents the abundance of archaeal genera in each sample of the corresponding category. Notable differences in archaeal population are those where the taxon is abundant in CM (cow and mouse) samples, and effectively undetected in the H- (cow and mouse) controls. The distribution and relative abundance of the archaeal genera in the study metagenomes are also available in Additional file 2

Consistent with the variation in the bacterial component of the microbiomes, we concurrently found notable differences in the relative abundances of the viral (Fig. 7, Additional file 4) and archaeal (Fig. 8, Additional file 5) components in CM and H samples of both cows and mice (Additional file 2). For instance, the CM mice fecal samples (MCMF) were enriched with higher relative abundances of *Siphovirus* (61.98%), *Mastadenovirus* (14.17%) and *Myovirus* (12.21%), and *Gammaretrovirus* (37.77%), whereas the CM mice mammary tissues (MCMMT) were predominated by *Ichnovirus* (21.90%), *Betaretrovirus* (15.45%), and *Macavirus* (12.69%) (Fig. 7, Additional file 2). In contrast, *Siphovirus* (61.98%), *Mastadenovirus* (14.17%) and *Myovirus* (12.21%) in H mice fecal samples (HMF), and *Gammaretrovirus* (36.12%), *Ichnovirus* (23.0%), *Betaretrovirus* (14.43%), *Macavirus* (13.11%) and *Rhadinovirus* (5.04%) in H mice mammary tissues (HMMMT) had higher relative abundances compared to the CM samples. The remaining viral genera in the CM and H samples of mice had relatively lower abundances (< 5.0%) (Fig. 7, Additional file 2).

The present microbiome study demonstrated significant differences (*p* = 0.012, Kruskal–Wallis test) among the archaeal community in the sample categories of both hosts (cow and mice) with mastitis at the genus level. The CM cow fecal metagenome (CCMF) was dominated by *Aeropyrum* (11.12%), *Ferroplasma* (3.81%), *Cenarchaeum* (3.72%), *Desulfurococcus* (3.31%), *Haloarcula* (2.90%), *Halobacterium* (2.77%), *Halogeometricum* (2.33%) and *Methanobrevibacter* (1.06%) genera whereas *Methanosphaerula* (33.05%), *Methanoculleus* (5.08%), *Methanoplanus* (2.54%) and *Pyrococcus* (2.54%) were identified as the top abundant archaeal genera in CM cows milk samples (CCMM) (Fig. 8, Additional file 7). Among the identified archaeal genera in the mastitis induced mice metagenomes, *Acidilobus* (23.74%), *Aciduliprofundum* (14.47%), *Archaeoglobus* (8.34%), *Aeropyrum* (6.93%), *Caldivirga* (5.12%), *Candidatus* (3.04%), *Cenarchaeum* (2.65%), *Desulfurococcus* (2.54%), *Ferroglobus* (2.51%), *Ferroplasma* (2.26%), *Halalkalicoccus* (2.26%), *Haloarcula* (2.01%), *Haloferax* (1.98%), *Halobacterium* (1.79%), *Halogeometricum* (1.67%), *Halomicrobium* (1.63%) in MCMF metagenome, and *Methanosaeta* (46.23%), *Methanoplanus* (22.64%), *Methanosphaerula* (%11.32%), *Methanosarcina* (3.77%), *Acidilobus* (1.89%), *Aciduliprofundum* (1.89%), *Methanobrevibacter* (1.89%), *Methanoculleus* (1.89%), and *Pyrococcus* (1.89%) in MCMMT metagenome were the most dominant genera (Fig. 8, Additional files 2, 7). Conversely in healthy mice, *Aciduliprofundum* (15.02%) in HMF, and *Archaeoglobus* (11.03%), *Halobacterium* (4.26%), *Halalkalicoccus* (3.01%), *Halogeometricum* (2.76%), *Haloquadratum* (2.51%), *Methanoculleus* (2.51%), *Halomicrobium* (2.01%) and *Ignicoccus* (2.01%) in HMMT were the most abundant archaeal genera. Although, the remaining archaeal genera detected in this study had a relatively lower abundance (< 1.0%), their abundance always remained higher in CM associated murine samples (Fig. 8, Additional files 2, 7).

### CM-associated genomic functional perturbations of microbiomes in murine mastitis

**Fig. 9:**
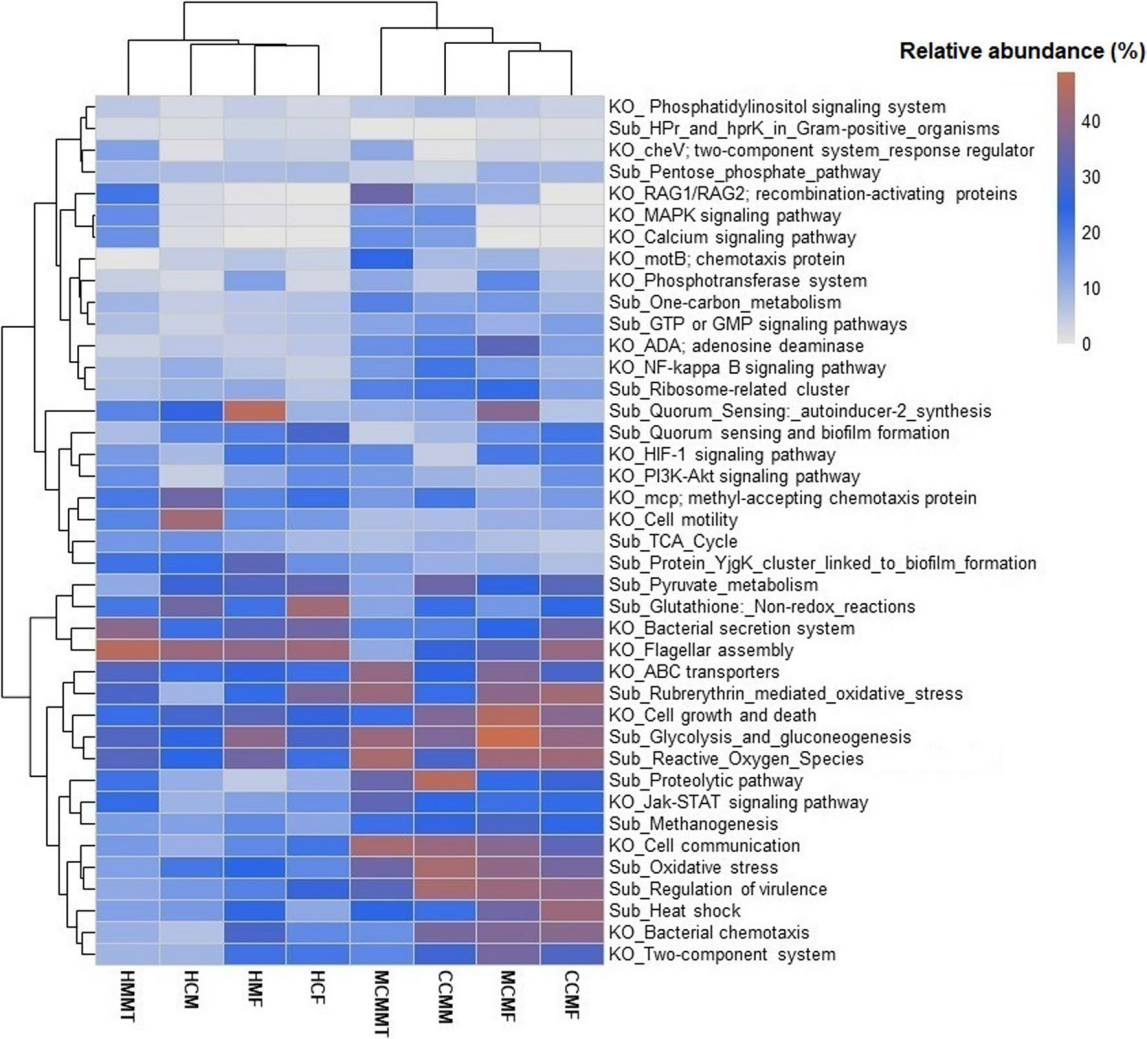
Functional annotation of the cow and mouse mastitis (CCMM, CCMF, MCMF, MCMMT) and healthy (HCM, HCF, HMF, HMMT) sample related sequences. Heatmap representing hierarchical clustering of the predicted KEGG Orthologs (KOs) and SEED subsystem (Sub) functional pathways of the microbiome based on the average relative abundances across eight metagenome groups (CCMM, CCMF, MCMF, MCMMT) and healthy (HCM, HCF, HMF, HMMT). The color bar (light blue to dark red) at the top right-hand of the heatmap represent the relative abundance of putative genes expressed as a value between 0 (lowest abundance) and 40 (highest abundance) in each sample category. The dark red color indicates the more abundant patterns, whilst light blue cells account for less abundant putative genes in that particular metagenome. More information on the predicted metabolic functional potentials of the microbiomes in the study metagenomes are also available in Additional file 8

The WMS data were further analyzed using an assembly-based hybrid method of MG-RAST 4.0 (MR) [33] to compare the genomic functional potentials of the microbiomes. In MR analysis, 90.29 million reads (43.15% of cleaned reads) mapped to putative genes with known protein functions after filtering against host associated reads (Data S1). We identified 154 differentially abundant KEGG orthologues; KOs (including MCMF = 149, HMF = 144, MCMMT = 55, HMMT = 109, CCMF = 142, HCF = 131, CCMM = 110 and HCM = 129) and 61 SEED functions (including MCMF = 59, HMF = 59, MCMMT = 44, HMMT = 56, CCMF = 59, HCF = 61, CCMM = 56 and HCM = 60) at different subsystem levels across the bovine and murine metagenomes (CM and H). By comparing the composition and relative abundances of the different Kos or SEED functions in the same KEGG pathway or SEED subsystem between CM- and H-metagenomes, we found significant differences (*p* = 0.003, Kruskal Wallis test) in their relative abundances indicating positive correlations with CM in both hosts (Fig. 9, Additional file 2). Moreover, by measuring the relative abunadances of these functional pathways between CM- and H-mice, our analysis revealed that bacterial chemotaxis (67.18%), primary immunodeficiency; ADA (32.11%), methanogenesis (29.16%), phosphotransferase system (17.88%) in CM-mice feces (MCMF), and glycolysis and gluconeogenesis (61.90%), reactive oxygen species; ROS (43.90%), cell-to-cell communication (43.15%), ABC transporters (40.25%), recombination-activating proteins; RAG1/RAG2 (34.21%), chemotaxis protein; motB (23.76%), Jak-STAT signaling pathway (32.23%) and one-carbon metabolism (18.75%) in CM-mice mammary tissues (MCMMT) were the predominantly enriched metabolic functions compared to their H counterparts (Fig. 9, Additional file 8). On the contrary, genes coding for quorum sensing: autoinducer-2 synthesis (46.81%) and HIF-1 signalling pathway (21.16%) in H-mice feces (HMF), and flagellar assembly (46.08%), bacterial secretion system (39.25%), and citrate synthase; gltA (15.61%) in H-mice mammary tissues (HMMT) were the enriched metabolic functions compared to the CM associated microbiomes. Moreover, remaining KOs and SEED modules identified in this study also varied in their relative abundances and had relatively lower abundance in H metagenomes (Fig. 9, Additional file 8).

### Relationships between predominant microbial species and their genomic functional potentials in murine mastitis

**Fig. 10:**
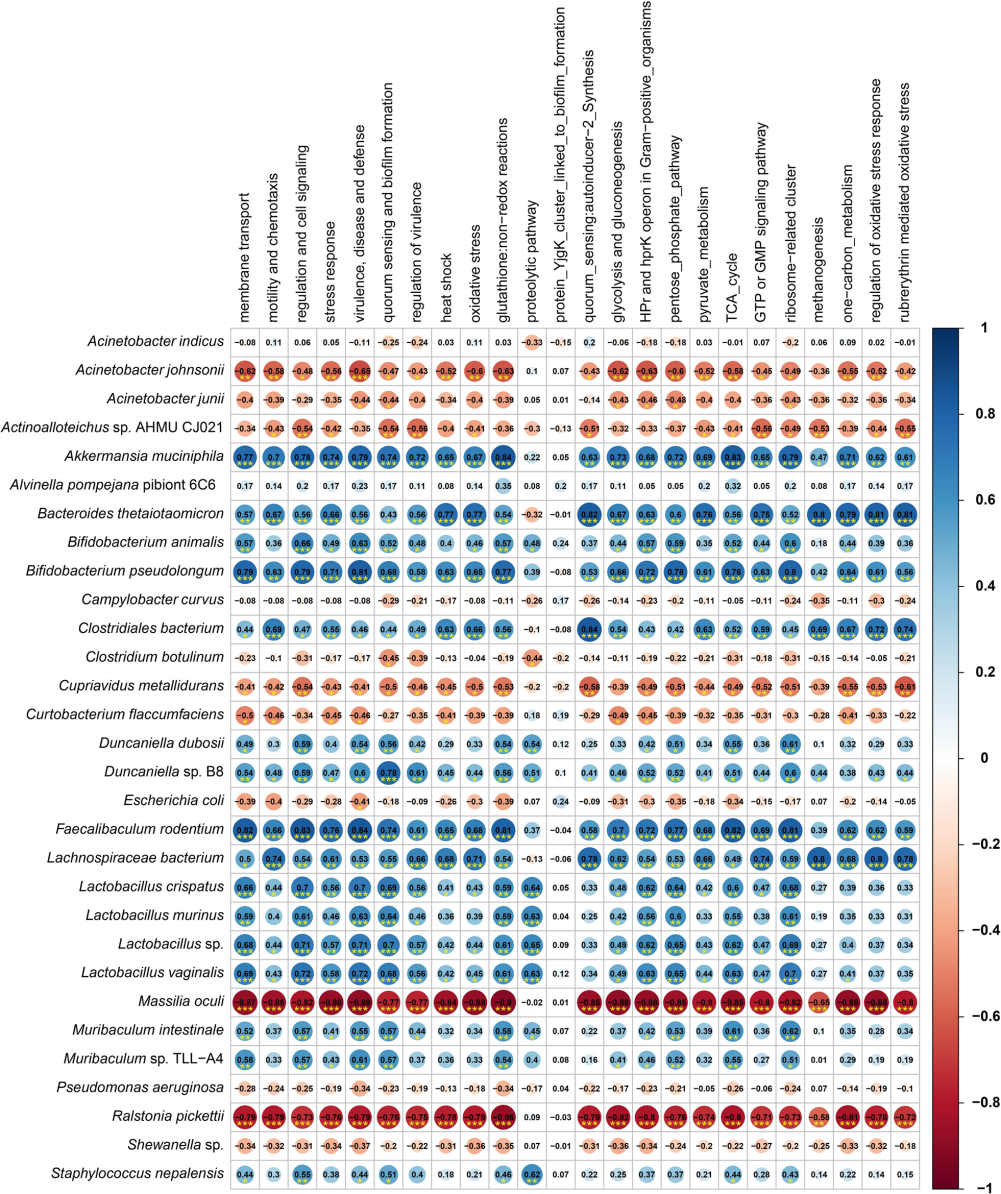
The correlation between top abundant 30 bacterial species and their genomic functional potentials at different levels of SEED subsystem in CM and H control metagenomes of both cows and mice. The scale bar at the right side of of the heatmap represent the Spearman’s correlation coefficient (r). Blue and red indicate positive and negative correlation, respectively. The color density, circle size, and numbers reflect the scale of correlation. *Significant level (*p < 0.05; **p < 0.01; ***p < 0.001)

In this study, *B. pseudolongum*, *Bacteroides thetaiotaomicron*, *D. dubosii*, *Duncaniella* sp. B8, *Faecalibaculum rodentium*, *L. bacterium*, *L. murinus* and *Muribaculum* sp. TLL-A4 had strongest positive correlations (Spearman’s correlation; r > 0.5, *p* < 0.01, Fig. 10) with different SEED functions including membrane transport, quorum sensing and biofilm formation, oxidative stress, rubrerythrin mediated oxidative stress, regulation of oxidative stress response, protein YjgK cluster linked to biofilm formation, virulence, diseases and defenses, proteolytic pathways, GTP or GMP pathways, methanogenesis, glycolysis and gluconeogenesis, and one-carbon metabolism (Fig. 10). Conversely, *C. botulinum*, *A. johnsonii* and *Actinoalloteichus* sp. AHMU revealed significant negative correlations (Spearman’s correlation; r ≥  − 0.4, *p* < 0.05) with most of the SEED modules (Fig. 10). Simultaneously, *A. muciniphila*, *B. pseudolongum*, *B. thetaiotaomicron*, *B. animalis*, *D. dubosii*, *Duncaniella* sp. B8, *F. rodentium*, *L. bacterium*, *L. murinus* and *Muribaculum* sp. TLL-A4 showed significantly higher positive correlations (Spearman’s correlation; r ≥ 0.6, *p* < 0.01) with KOs like cheV, motB, ABC transporters, phosphotransferase and two-component systems, ADA, RAG1/RAG2 and mcp (Additional file 9). Consistent with SEED functions, *C. botulinum*, *M. oculi*, *A. johnsonii*, *A. junii*, *Actinoalloteichus* sp. AHMU and *H. cinaedi* displayed negative correlations (Spearman’s correlation; r ≥  − 0.4, *p* < 0.05) with most of the KOs identified in this study (Additional file 9).

## Discussion

Metagenomics is a powerful tool for shedding light on microbiome signature and concurrent genomic features associated with the process of animal disease. Our cow-to-mice induced mastitis model developed in this study clearly indicated that microbiome dysbiosis and concurrent genomic functional perturbations are associated with mammalian mastitis. In this study, more than 8.5 million metagenomic reads were assigned to taxonomic composition which is thought to be sufficient enough to capture maximum microbial richness and corroborated with our previous studies [7, 8, 12]. Several previous studies [34, 35] reported that in shotgun metagenome (WMS) study, more than 90% sequence reads may come from host DNA supporting our present findings. One of the hallmark findings of this study is the identification of several new bacterial species associated with both bovine (e.g., *L. crispatus*, *N. pseudobrasiliensis*, *L. vaginalis*, *C. difficile*, *R. insidiosa*, *B. pseudolongum*, *Muribaculum* sp., *Duncaniella* sp., *D. dubosii*., and *Actinoalloteichus* sp.) (Fig. 4) and murine (e.g., *Muribaculum* sp., *Duncaniella* sp., *M. intestinale*, *B. animalis*, *D. dubosii*, *A. muciniphila*, *L. crispatus*, *B. pseudolongum*, *L. murinus*, *M. oculi*, *R. pickettii*, *C. flaccumfaciens*, and *C. acnes*) mastitis (Fig. 5). So far, none of these species have been reported to be associated with bovine and murine CM and other lactating mammals.

### Microbial dysbiosis and host-tropism occurs in mastitis pathogenesis

In the present study, the fecal microbiota of cows and mice differed significantly at the species level. The prevalence of these microbiotas significantly varied across the CM and H metagenomes of both the hosts. The association of *P. aeruginosa*, *K. oxytoca*, *P. dispersa*, *E. faecalis*, *S. aureus*, *A. baumannii*, *A. johnsonii*, *K. pneumoniae* and *E. coli* in bovine CM corroborated with our previous studies [7, 8, 12]., However, the relative abundance of these species remained substantially higher in this study than what we reported in earlier studies [7, 8, 12]. Consistent with these findings of taxonomic discrepencies in microbiome signature and abundances between two respective sample categories (CM vs. H). We also observed the distinct changes in the genomic functional features of the microbiomes in the two hosts (cow and mice) and sample categories between groups (CM and H) (Fig. 9).

The findings of the present study revealed that majority of the species of *Lactobacillus* were identified with higher abundances in the CM fecal samples of both cows and mice, which support their positive association with CM as also reported previously in a number of studies [24, 36, 37]. Lactic-acid bacteria of the genera *Lactobacillus*, *Bifidobacterium*, *Bacillus* and *Enterococcus* are commonly found in the human [38], avian [39] and animals [37, 40] gut. Different species and/or strains of these genera are good candidates to compete with pathogens for mammary gland colonization [41]. However, no direct scientific evidence is currently available regarding the typical role of these microbes in the pathophysiology of mastitis. *K. pneumoniae* and *N. pseudobrasiliensis* are the emerging opportunistic environmental pathogens, and transmission of these pathogens to the mammary glands might occur from feces and bedding materials [7, 8, 42]. *E. faecalis*, one of the major pathogens of bovine mastitis, generally present in organic bedding materials and feces being opportunistic invaders of mammary glands [17, 30]. Additionally, a novel species of *Actinobacteria* phylum, *Actinoalloteichus* sp. was predominantly found only in CM cows milk samples (not in mouse samples), which argues against *Actinoalloteichus* sp. as the causative agent of murine mastitis. However, *Actinoalloteichus* has recently been detected as the most abundant genus in milk [43] supporting our present findings. *P. dispersa* can cause a variety of infections in immunocompromised dairy cows [8, 10], however, *P. melaninogenica*, a member of the normal microbiota of the human breast tissue [44], has never been described in cow feces and milk until now. Notably in this study, microbial communities originating from H-control samples have differed from those of CM samples. For instance, the detection of *P. aeruginosa*, *E. faecalis*, *P. dispersa*, *A. baumannii* and *P. melaninogenica* at higher levels within normal H fecal and milk is intriguing (Fig. 4). *Pseudomonas* is an animal skin microbe, which can colonize the udders and/or quarters from contaminated water and fecal sources [12, 45] and thus, potentially act as opportunistic pathogen by causing intramammary infections [8, 10]. This study therefore revealed a close association between the gut microbiome and milk microbes in the pathogenesis of bovine CM as also reported previously [7, 8, 46]. Additionally, *M. oculi* which was predominantly identified in the mammary tissue of both CM and H mice had significantly lower abundance in fecal and milk samples of cows. This soil bacterium has recently been detected in human clinical specimen [47], however, this species has not been reported in either cows or mice.

Notably, we detected some organisms that are indicative of the mastitis in GF mice (Fig. 5). Our results showed that CM mice sample groups had the highest number of indicator bacterial species. For example, *P. durus*, *S. maltophilia*, *P. russatus* and *P. polymyxa* were identified as good indicators of the murine CM (IndVal ≥ 0.8, *p* = 0.002). This high indicator value suggested that these species were found in most of the samples from CM mice and were comparatively less abundant in H mice samples (Fig. 5). Moreover, different species of *Paenibacillus* have potential contributions to maintaining mammary gland homeostasis [17], and we therefore suggest that association of *Paenibacillus* and *Pontibacter* in mammalian mastitis could be as opportunistic pathogens. In addition, the multidrug resistant bovine CM pathogen, *S. maltophilia* [17, 48] was found with higher relative abundances in CM mice fecal samples indicating its potential role in the induction of mastitis in GF mice. We furhter found that different species of *Clostridia*, *Bacteroida*, *Actinobacteria*, *Flavobacteriia* and *Betaproteobacteria* had strong co-occurrence and positive correlation as indicator species of murine mastitis. In this study, *P. thermosuccinogenes*, *P. xylanivorans, M. intestinale*, *B. uniformis*, *P. polymyxa*, and *B. animalis* showed more connections and overlap suggesting their cooperative or syntrophic interactions during the pathogenesis of murine mastitis (Fig. 6). This is further supported by the higher relative abundance of these microbial taxa in CM mice samples.

### Multi-microbial pathogenesis favors mastitis through metabolomic cross-talk

Mastitis is a polymicrobial (including bacteria, viruses, archaea) disease where both archaea and viruses are considered as traditionally neglected microbes. Unlike the bacteria, the diversity, composition, and the relative abundances of viruses (Fig. 7) and archaea (Fig. 8) remained much lower in this metagenome study. Our previous studies [7, 8, 10, 12] also reported the concurrent occurrence of archaea and viruses in bovine mastitis highlighting the notion that mastitis is a multietiological disease [8, 10, 29, 49]. Archaea are major colonisers of rumen or the intestinal tracts of animals or humans [50], and thus, their abundance in milk always remains much lower. Recently, majority of the archaeal genera we detected, have been identified from a diverse group of samples including the fecal sample of cattle [51], canine [52] and swine [53], bovine milk [7, 8] and saliva of human [54] supporting our present findings. Mastitis is the result of direct interactions of bacteria with hosts (host–pathogen interactions) under immunosuppression or stressed conditions (when the cow suffers from a severe negative energy balance at the onset, in-and-around lactation, and other environmental stress e.g., heat stress). During the progression of bacterial mastitis, viruses jump into, and reach the site of inflammation in the mammary tissues, and triggers pathogenic pathways by the lysis of macrophages [55]. This phagocytic macrophage storming further aggravates the pathogenesis and creates a micro-aerobic/anaerobic condition which ultimately favors the archaeal growth [56, 57]. Therefore, both viruses and archaea may act as a predisposing factor as well as a primary etiological agent for more severe and prolonged mastitis [7, 8, 10, 12].

In this study, we also found alteration in relative abundances of some important predicted genomic functions among different sample groups of CM and H microbiomes (Fig. 9). The metabolic features identified in the same KEGG pathway or SEED subsystem varied across mastitis samples in both cows and mice, suggesting their possible association in the early colonization and disease progression [8, 17]. Higher abundance of genes associated with bacterial chemotaxis, two-component system, GTP or GMP, NF-kappa-B and Jak-STAT signalling pathways in CM microbiomes of both hosts suggests their potential roles in mastitis through several complex biologic processes including immune disorders, cell differentiation, migration, proliferation, expression of many cytokines, quorum-sensing, microbial group behaviours and oxidative stress mediators, which likely accounts for the high systemic pathogen burden [7, 58–60]. Moreover, the higher abundance of ROS in CM-samples may contribute to the development of oxidative stress and inflammatory response [61] to further aggravate the pathogenesis of mastitis. Besides, quorum sensing, biofilm formation, glutathione non-redox reactions, and methyl-accepting chemotaxis genes that were predominantly identified in CM-pathogens play an important role in many opportunistic bacterial infections [8, 30]. Conversely, the PI3K-Akt pathway related genes remained highly expressed in H-microbes, and this pathway plays a critical role in the regulation of cell growth, proliferation, survival, motility, differentiation, angiogenesis, and metabolism [62].

The observed differences in microbiome composition and their corresponding genomic functional properties are considered to be the co-selection factor for mammary gland pathogenesis. Notably, the enriched consortia of *B. pseudolongum*, *B. thetaiotaomicron*, *D. dubosii*, *Duncaniella* sp. B8, *F. rodentium*, *L. bacterium*, *L. murinus*, *Muribaculum* sp. TLL-A4, *A. muciniphila* and *B. animalis* species had the strongest positive correlations with most of the KEGG and SEED functional pathways, indicating these microbial members could play potential roles in the pathophysiology of mastitis (Fig. 10).

### Cow-to-mouse model may be a useful protocol for mastitis diagonosis, curative and preventive studies

Laboratory animals like mice provide an effective experimental model for understanding the underlying mechanisms of host–microbe interactions [5, 63]. The mouse mastitis model seems to be a good model to study bovine mastitis compared to other laboratory animals for ease of handling, controlled environments, and low maintenance cost [64]. Despite restoring the common mastitis syndromes in both cows and mice, we observed marked differences in microbiome composition and relative abundances irrespective of the sample categories. Therefore, the findings of this study imply that mastitis is not solely caused by the resident microbiota of the mammary gland and/or its secretory product milk but can also be impacted by the alterations of gut microbiota and their genomic functional potentials. Our results highlight the paramount importance of existing entero-mammary pathways through which gut and milk microbiota (from CM host only) could transfer to induce mastitis. The results of the present study corroborated with the previous findings of Ma et al. [5] who reported that bovine mastitis is not necessarily a local infection of mammary glands, rather can be caused by a dysfunctional intestinal microbiota. Taken together, the core microbiota identified in the present study distinguished gut and mammary microbiota not only with different sample sources, but also with the health and/or disease state of mice (CM vs. H). Therefore, restoration of gut and mammary gland ecosystem function, for example the mastitis-associated pathways identified in this study, could possibly serve as an effective therapeutic target for bovine mastitis, which may deserve further validation in a larger representative cohort of dairy cows. However, further research is needed to understand the mechanism that allows gut and environmental microorganisms to invade the mammary glands and/or quarters and their relationships with mammalian hosts immune system.

## Conclusions

The omics approach employed to study mastitis pathogenesis clearly showed microbiome dysbiosis. Species bias was dependent on the host and the predicted genomic functional features in CM hosts were significantly different from the H control counterparts. Our results show that few mastitis-associated microbial taxa and/or genomic functions were shared between diseased (CM) cows and mice regardless of conservation of mastitis syndromes. Cow-to-mouse microbiota transplantation protocols for induction of mastitis might be useful for further molecular studies of mastitis, which will ultimately improve the prevention and treatment strategies in both human and animal species. Taken together, a high-level abundance of the dominant and indicator microbial communities, associated genomic functional potentials, and their simultaneous correlations with the pathogenesis of mammary glands are considered to be driving factors for the mammalian mastitis.

## Methods

### Dairy cow selection and sampling

Twelve (n = 12) Holstein crossbred cows (including CM = 7 and H = 5) from seven dairy farms in Dhaka district (23.81 N, 90.41 E) of Bangladesh were used as the donors of fecal and milk samples. The age, parity and lactation of the cows ragend from 2.5 to 6 years, 1 to 5, and 7 to 45 days, respectively (Additional file 1). The cows gave birth randomly throughout the year (no particular control breeding), were milked once daily with their calves used for stimulating milk letdown. The cows were fed on rice straw, cut-and-carry grasses and milling by-products as concentrate (crashed rice and/or sometimes mustard oil cake) with limited grazing [65]. California mastitis test (CMT) was employed initially to diagnose CM following a previously published protocol [2], and manufacturer’s instruction (CMT^®^, Original Schalm reagent, ThechniVet, USA). In brief, about 2 mL of milk sample was squirted in each cup of mastitis paddle, and an equal volume (2 mL) of CMT reagent was added to the cups. The reactions were developed within 20 s in positive samples and was categorized into five grades based on gel formation (Scandinavian scoring system) in the reaction mixture viz. 0 (negative), T (trace, possible infections), 1 (weak positive), 2 (distinct positive), and + 3 (strong positive) [2]. The cows having a CMT score of ≥ 2 along with gross visible signs of mastitis were designated as CM cows [2]. A total of 12 milk samples (including CM = 7 and H = 5) were collected from lactating cows. Approximately, 15–20 mL of milk from each cow was collected in a sterile falcon tube during the morning milking (9.0–11.0 am) with an emphasis on pre-sampling disinfection of teat-ends and hygiene during milk sampling [2]. Simultaneously, we collected 12 fresh fecal samples (including CM = 7 and H = 5) from the selected cows under hygienic condition. Aprroximately, 5–10 g of fecal sample was collected through the rectum of each cow wearing a hygienic disposable plastic glove. No lubricants were used during sample collection. Collected fecal samples were then mixed-well and placed in a sterile falcon tube after proper labelling (15 mL) [5]. For either the CM or H group, both milk and fecal samples were freshly collected, transported to the laboratory keeping in an ice box (at 4 °C temp), the content was thereafter processed and divided into aliquots. A portion of the processed samples proceeded to FMT and MMT immediately after collection, and rest of the aliquoted samples were stored at − 80 °C for metagenomic DNA extraction.

### Microbiota transfer experiments

For the FMT and MMT procedures, forty (n = 40) timed-pregnant (at Day 17 of breeding) Swiss albino mice were procured from the ICDDR’B, Dhaka, Bangladesh. The mice were randomly divided into four groups: Group-I (FMT from CM cows, n = 10) Group-II (FMT from H cows, n = 10), Group-III (MMT from CM cows, n = 10) and Group-IV (MMT from H cows, n = 10). The mice were challenged on the same day of sampling. At the day of challenge (Day 17 after mating), 0.5 g fecal sample obtained from each of the CM and H cow was resuspended with twice the fecal volume of sterile physiological saline. After thorough mixing and resting (to minimize the number of bacteria lost), the supernatant was collected, and FMT was performed by a single oral administration of 1 g/kg fecal suspension to each mouse of Group-I and Group-II [66]. Like wise, 10 mL whole milk tubes (from both CM and H cows) were centrifuged at room temperature for 10 min at 5000× *g* [67]. After centrifugation, both the cream layer and supernatant liquid were removed. The pellet was resuspended to the initial sample volume of sterile physiological saline, and MMT was performed by a single oral administration of 0.5 mL of milk suspension to each mouse of Group-III and Group-IV [68]. The mice were housed in GF environment on a 12 h light/dark cycle with unlimited access to food and water. In order to prevent cross-contamination of gut microbiota, the four groups of mice were physically separated into different GF isolators after challenge. Moreover, each mouse was housed in a separate cage with safe distance apart within each of the individual GF isolator, so as to prevent any island effects [66, 69]. At the end of Day 27 of mating (10 days of challenge), the mice were sacrificed, and fresh fecal samples, mammary and gut (colon) tissues were collected. The collected fecal samples were then mixed with freshly prepared phosphate buffered saline (PBS), and finally stored at − 80 °C until further processing and DNA extraction.

### Histopathological analysis

Collection of milk and subsequent milk somatic cell count (SCC) was not feasible in challenged mice, therefore histopathological examination was performed to assess the alterations and inflammatory changes of mammary gland and colon tissues during mastitis. The mammary gland and colon tissues were dissected from mice, kept in glass tubes, and fixed in 4% paraformaldehyde (PFA) (Sigma-Aldrich) for 24 h at 4 °C. Paraffin-embedded tissues were cut on a Leica Rotary Microtome (Leica Microsystems), placed onto SuperFrost Plus slides, and dried overnight at 37 °C. Sections were deparaffinized with xylene, and gradually rehydrated through graded alcohols for staining with standard hematoxylin and eosin (H & E staining) sectioned [70]. To assess the degree of tissue injury to the mammary gland and colon, we used the Chiu Scoring System [71] in a blinded manner, where the number of inflammatory cells was counted in 12 randomly selected fields from each slide at a magnification of 400× . The slides were observed for severe, diffuse interstitial and/or alveolar infiltrate of inflammatory cells, focal to multifocal areas of tissue damage, epithelial abnormalities and extensive necrotic areas in mastitic mice [70]. The degree of necrosis in mammary gland tissues was scored on a scale of 0–3 (normal 0, mild 1, moderate 2, severe 3). The degree of colon injury was scored as grades 0 (normal mucosa), 1 (development of subepithelial spaces at villus tips), 2 (extension of the subepithelial space with moderate lifting of the epithelial layer), 3 (massive epithelial lifting with a few denuded villi), 4 (denuded villi with exposed capillaries), and 5 (disintegration of the lamina propria, ulceration, and hemorrhage) [66, 70]. The slides were assessed under an Olympus BX51 upright microscope (40× objective), and finally images were collected using an Olympus DP73 camera through cellSens entry software (Olympus Corporation, Japan), and visualized using image J software (https://imagej.nih.gov/ij/).

### Genomic DNA extraction and whole metagenome sequencing

Total genomic DNA from six milk (CM = 3, H = 3), six mammary tissue (CM = 3, H = 3), and 12 fecal (CM = 8, H = 4, from both cow and mice) samples (Data S1) was extracted using Maxwell 16 automated DNA extraction platform (Promega Corporation, Madison, WI 53711-5399, USA). In brief, DNA from fecal (400 µL fecal suspension) and milk (400 µL of whole milk) samples was extracted using Maxwell^®^ 16 FFS Nucleic Acid Extraction Kit [7] and Maxwell^®^ 16 FFPE Plus LEV DNA Purification Kit [72], respectively. For DNA extraction from fecal samples, 80 mg fecal material was added to 400 μL lysis buffer and mixed thoroughly by vortexing to make a suspension. The Maxwell^®^ 16 Tissue DNA Purification Kit (Promega Corporation, Madison, WI 53711-5399, USA) was used for DNA extraction from mammary tissue (50 mg) samples following previously published protocol [73]. DNA quantity and purity were determined using NanoDrop ND-2000 spectrophotometer (ThermoFisher, USA) by measuring 260/280 absorbance ratio. Libraries for shotgun WMS were prepared with Nextera XT DNA Library Preparation Kit [74] according to the manufacturer’s instructions, and paired-end (2 × 150 bp) sequencing was performed using a NovaSeq 6000 sequencer (Illumina Inc., USA). Our metagenomic DNA yielded 517.14 million reads with an average of 21.55 million (maximum = 22.96 million, minimum = 18.76 million) reads per sample (Data S1).

### WMS data processing and microbiome analysis

The generated FASTQ files were concatenated and filtered through BBDuk (available from https://sourceforge.net/projects/bbmap/) with options k = 21, mink = 6, ktrim = r, ftm = 5, qtrim = rl, trimq = 20, minlen = 30, overwrite = true [75] to remove Illumina adapters, known Illumina artifacts, and phiX. Any sequence below these thresholds or reads containing more than one ‘N’ were discarded. The WMS data were analyzed using both open-source cloud-based metagenomic mapping-based and assembly-based hybrid methods of IDSeq [32] and MG-RAST 4.0 (MR) [33], respectively. IDseq is an open-source cloud-based pipeline for taxonomic assignments with NTL (NTL; nucleotide alignment length in bp) ≥ 50 and NT % identy ≥ 90. In IDSeq analysis, a ‘target’ genome library was constructed containing all prokaryotic sequences from the NCBI Database. The WMS reads were then aligned against the target libraries using the very sensitive Bowtie 2 algorithm [76], and filtered with Trimmomatic [77] to remove the reads aligned with the cattle (bosTau8), mouse (GRCm39) and human (hg38) genomes. The raw sequences were simultaneously uploaded to the MR server with properly embedded metadata, and were subjected to quality filtering with dereplication, host DNA removal (filtering against the set reference genome of both cattle, mouse and human), and finally screening for metabolic functional assignment. We used minimum identity of ≥ 90% for metabolic functional analysis through KEGG pathways and SEED subsystems in the MR pipeline. In IDSeq pipeline, 4.06% reads (of total cleaned reads) mapped to the target reference genomes whereas in MR pipeline 43.15% reads mapped to different known protein functions in KEGG pathways and SEED subsystems after filtering the cow, mouse and human genomes (Additional file 2).

Alpha diversity (diversity within samples) was estimated using the observed species, Chao1, Shannon and Simpson diversity indices [78] for IDSeq read assignments and counts. To visualize differences in microbiome diversity, a principal coordinate analysis (PCoA) based on the Bray–Curtis distance method [79], and non-metric multidimensional scaling (NMDS) measured by weighted-UniFrac distance on IDSeq data were performed through Phyloseq R package, version 4.1 [80]. Indicator species specific to a given sample group in mouse model (having ≥ 1000 reads assigned to a taxon) were identified based on the normalized abundances of species using the R package, indicspecies [81], and the significant indicator value (IndVal) index was calculated by the 999-permutation test. Data were processed through the Phyloseq R package, visualized by using ggplot2 [82]. The Venn diagrams representing taxonomic composition were generated through a stand-alone software tool; FunRich (http://www.funrich.org/). The differences in the microbiome abundances (Z-scores) across the study metagenomes was calculated using Spearman’s correlation test. Z-scores were calculated to construct heatmap for showing the relative abundance of the microbes in each sample group with the formula: Z = (x − μ)/σ, where x is the relative abundance of microbes in each sample category, μ is the mean value of relative abundances of microbes in all samples, and σ is the standard deviation of relative abundances [83]. Finally, a heatmap of normalized Z-scores (from 0 to 40) of relative abundances of bacterial populations was produced. In addition, the Spearman’s correlation coefficient and significance tests for the indicator species were performed using the R package Hmisc (https://cran.r-project.org/web/packages/Hmisc/index.html). A correlation network was constructed and visualized with Gephi (ver. 0.9.2) (https://gephi.org/) to explore the co-occurrence patterns of the indicator species.

### Metabolic functional potential analysis

The genomic functional profile of the microbiomes was annotated according to the Kyoto Encyclopedia of Genes and Genomes (KEGG) Orthology [84], and SEED subsystem [85] databases in MR pipeline using a “Best Hit Classification” method. The functional mapping was performed with the partially modified set parameters (*e-*value cutoff: 1 × 10^−30^, min. % identity cutoff: ≥ 90%, and min. alignment length cutoff: 20) of the MR server [86]. We also investigated the relationships between the relative abundances of the predominating bacterial species and their genomic functional potentials in the pathogenesis of murine mastitis after renormalization and permutations.

### Statistical analyses

The pair-wise non-parametric Kruskal–Wallis rank sum test was used to evaluate differences in the relative percent abundance of the microbial taxa and differentially abundant SEED or KEGG functions (at different levels) in CM and H animal (cow and mice) groups. Indicator species analysis calculated an IndVal which was the product of the relative frequency and relative abundance of a species in a cluster. To test the significance of the IndVal, *p* values were calculated with 100 iterations, where in each iteration, the sample groupings were randomly assigned and an IndVal determined. The *p* values for the IndVal calculation were corrected for multiple comparisons using the false discovery rate correction [87]. To explore the relationship among the relative abundance of indicator species, we calculated Spearman’s rank correlation coefficients using the ‘multtest’ package in R [88], and the correction was made using Benjamini–Hochberg FDR. In the co-occurrence network, each node represents one species and each edge stands for correlation between the species abundances [87]. Spearman’s correlation among the top abundant 30 bacterial species, and their genomic metabolic functions (SEED and KEGG functional pathways were performed in Hmisc and corrplot R packages as described above (Methods).

## Supplementary Information

**Additional file 1.** Study information (animal information, location, breeds, lactation, and parity).

**Additional file 2.** Information on metagenomic data, microbial structure, composition and relative abundances in each metagenome.

**Additional file 3.** Taxonomic structure of microbiomes identified in different sample groups.

**Additional file 4.** Taxonomic composition of viruses.

**Additional file 5.** Taxonomic composition of archaea.

**Additional file 6.** Taxonomic information of top seventy bacterial species identified in different metagenomic groups.

**Additional file 7.** Archaeal genera detected (with their relative abundances) in different metagenomes.

**Additional file 8.** Changes in metabolic functional profile in the study metagenomes.

**Additional file 9.** The correlation between predominantly abundant bacterial species and their genomic functional potentials in different KEGG orthologues (KOs) in CM and H control metagenomes of both cows and mice.

## Data Availability

The sequence data and related metadata reported in this paper are available in the SRA repository of the NCBI database under BioProject Accession ID of PRJNA753312. Supplementary information supporting the findings of the study are available in this article as Additional files 1, 2, 3, 4, 5, 6, 7, 8 and 9.

## Abbreviations

FMT: Fecal microbiota transplantation
MMT: Milk microbiota transplantation
GF: Germ free
CM: Clinical mastitis
H: Healthy
WMS: Whole shotgun metagenomic sequencing
CCMF: Cow clinical mastitis feces
HCF: Healthy cow feces
CCMM: Cow clinical mastitis milk
HCM: Healthy cow milk
MCMF: Mouse clinical mastitis feces
HMF: Healthy mouse feces
MCMMT: Mouse clinical mastitis mammary tissue
HMMT: Healthy mouse mammary tissue
