# Supplementary material for: Induction of mastitis by cow-to-mouse fecal and milk microbiota transplantation causes microbiome dysbiosis and genomic functional perturbation in mice
Source: Anim Microbiome. 2022 Jul 6;4:43. doi: 10.1186/s42523-022-00193-w (PMC9258091; doi:10.1186/s42523-022-00193-w)
Supplement: Supplementary file 1 — Additional file 1. Study information (animal information, location, breeds, lactation, and parity). [file 42523_2022_193_MOESM1_ESM.docx]

**Additional file 1:** Study information (animal information, location, breeds, lactation, and parity). A total of 12 lactating crossbred cows including 7 clinical mastitis (CM), and 5 healthy (H), and 12 pregnant swis albino mice (germ free) including 7 CM, and 5 H were selected for the study.

| Cow ID | Sample ID | Health Status | Farm Location | GIS (Longi-/Latitude) | Breeds | Age | Lactation (Days after calving/challenge) | Parity |
| --- | --- | --- | --- | --- | --- | --- | --- | --- |
| Cow 1 | G1CM1 | CM | Dhaka | 23.81 N, 90.41 E | XHF | 3 yrs | 07 | 1 |
| Cow 2 | G1CM2 | CM | Dhaka | 23.81 N, 90.41 E | XHF | 5 yrs | 25 | 2 |
| Cow 3 | G1CM4 | CM | Dhaka | 23.81 N, 90.41 E | XHF | 4 yrs | 17 | 2 |
| Cow 4 | G1CM5 | CM | Dhaka | 23.81 N, 90.41 E | XHF | 3.5 yrs | 41 | 1 |
| Cow 5 | G1H2 | H | Dhaka | 23.81 N, 90.41 E | XHF | 2.5 yrs | 13 | 1 |
| Cow 6 | G1H1 | H | Dhaka | 23.81 N, 90.41 E | XHF | 5 yrs | 45 | 3 |
| Cow 7 | G2CM2 | CM | Dhaka | 23.81 N, 90.41 E | XHF | 5 yrs | 29 | 3 |
| Cow 8 | G2CM3 | CM | Dhaka | 23.81 N, 90.41 E | XHF | 6 yrs | 15 | 4 |
| Cow 9 | G2CM6 | CM | Dhaka | 23.81 N, 90.41 E | XHF | 3 yrs | 11 | 1 |
| Cow 10 | G2H1 | H | Dhaka | 23.81 N, 90.41 E | XHF | 4.5 yrs | 23 | 3 |
| Cow 11 | G2H3 | H | Dhaka | 23.81 N, 90.41 E | XHF | 5 yrs | 41 | 3 |
| Cow 12 | G2H6 | H | Dhaka | 23.81 N, 90.41 E | XHF | 6 yrs | 33 | 4 |
|  | |  | | | | | | |
| Mouse 1 | G1M2CM | CM | Dhaka | 23.81 N, 90.41 E | Swiss albino | 75 dyas | 10 days of challenge | NA |
| Mouse 2 | G1M3CM | CM | Dhaka | 23.81 N, 90.41 E | Swiss albino | 75 dyas | 10 days of challenge | NA |
| Mouse 3 | G1M4CM | CM | Dhaka | 23.81 N, 90.41 E | Swiss albino | 75 dyas | 10 days of challenge | NA |
| Mouse 4 | G1M5CM | CM | Dhaka | 23.81 N, 90.41 E | Swiss albino | 75 dyas | 10 days of challenge | NA |
| Mouse 5 | G1M1H | H | Dhaka | 23.81 N, 90.41 E | Swiss albino | 75 dyas | 10 days of challenge | NA |
| Mouse 6 | G1M2H | H | Dhaka | 23.81 N, 90.41 E | Swiss albino | 75 dyas | 10 days of challenge | NA |
| Mouse 7 | G2M2CM | CM | Dhaka | 23.81 N, 90.41 E | Swiss albino | 75 dyas | 10 days of challenge | NA |
| Mouse 8 | G2M5CM | CM | Dhaka | 23.81 N, 90.41 E | Swiss albino | 75 dyas | 10 days of challenge | NA |
| Mouse 9 | G2M6CM | CM | Dhaka | 23.81 N, 90.41 E | Swiss albino | 75 dyas | 10 days of challenge | NA |
| Mouse 10 | G2M1H | H | Dhaka | 23.81 N, 90.41 E | Swiss albino | 75 dyas | 10 days of challenge | NA |
| Mouse 11 | G2M2H | H | Dhaka | 23.81 N, 90.41 E | Swiss albino | 75 dyas | 10 days of challenge | NA |
| Mouse 12 | G2M3H | H | Dhaka | 23.81 N, 90.41 E | Swiss albino | 75 dyas | 10 days of challenge | NA |

**Table S2:** Taxonomic structure of microbiomes identified in different sample groups.

| **Taxonomic ranks** | **Metagenome groups and number of taxa detected** | | | | | | | |
| --- | --- | --- | --- | --- | --- | --- | --- | --- |
|  | **CCMF** | **HCF** | **CCMM** | **HCM** | **MCMF** | **HMF** | **MCMMT** | **HMMT** |
| Bacterial species (n = 2191) | **1731** | | **618** | | **1065** | | **115** | |
|  | 1590 | 979 | 592 | 79 | 853 | 561 | 69 | 77 |
| Viral genera (n = 94) | 80 | 63 | 13 | 27 | 69 | 56 | 20 | 21 |
| Archaeal genera (n = 60) | 60 | 60 | 16 | 49 | 60 | 60 | 23 | 26 |

| **Table S3: Taxonomic information of top seventy bacterial species identified in different metagenomic groups.** | | | | | | | | | | |
| --- | --- | --- | --- | --- | --- | --- | --- | --- | --- | --- |
| **Phyla** | **Order** | **Species** | **CCMF** | **HCF** | **CCMM** | **HCM** | **MCMF** | **HMF** | **MCMMT** | **HMMT** |
| Proteobacteria | Pseudomonadales | *Pseudomonas aeruginosa* | 21.001 | 32.7 | 2.0131 | 40.352 | 0.0001 | 3.2 | 2.9 | 8.738 |
| Firmicutes | Lactobacillales | *Lactobacillus crispatus* | 12.102 | 0.008 | 0 | 0 | 2.873 | 1.798 | 0 | 0 |
| Proteobacteria | Enterobacterales | *Klebsiella oxytoca* | 10.324 | 2.9 | 20.68 | 4.0006 | 0.946 | 1.012 | 0 | 0.41 |
| Firmicutes | Lactobacillales | *Enterococcus faecalis* | 10.02 | 30.052 | 0.00036 | 0 | 5.0068 | 2.0201 | 0.59 | 0.41 |
| Proteobacteria | Burkholderiales | *Nocardia pseudobrasiliensis* | 5.002 | 2.0022 | 8.87 | 3.046 | 0 | 0 | 0.88 | 0.82 |
| Firmicutes | Lactobacillales | *Lactobacillus vaginalis* | 4.8543 | 0.003 | 0 | 0 | 0.934 | 0.961 | 0 | 0 |
| Firmicutes | Clostridiales | *Clostridioides difficile* | 4.231 | 0.77 | 1.7 | 0.0006 | 0.027 | 0.01 | 0.295 | 0 |
| Proteobacteria | Pseudomonadales | *Ralstonia insidiosa* | 4.005 | 2.005 | 2.5001 | 0.0297 | 0 | 0 | 0.59 | 0 |
| Actinobacteria | Bifidobacteriales | *Bifidobacterium pseudolongum* | 3.08 | 0.042 | 0.0003 | 0.0004 | 2.151 | 1.649 | 0 | 0 |
| Bacteroidetes | Bacteroidales | *Muribaculum sp.* | 2.354 | 0 | 0.001 | 0.0028 | 38.301 | 26.738 | 1.77 | 0 |
| Bacteroidetes | Bacteroidales | *Duncaniella sp.* | 2.325 | 0.576 | 0 | 0.0003 | 10.168 | 7.893 | 1.77 | 0.82 |
| Bacteroidetes | Bacteroidales | *Duncaniella dubosii* | 2.008 | 0 | 0.0005 | 0.0007 | 7.141 | 4.71 | 0.885 | 0.82 |
| Firmicutes | Erysipelotrichales | *Faecalibaculum rodentium* | 1.7801 | 0.169 | 0 | 0.0003 | 1.859 | 1.288 | 0 | 0 |
| Bacteroidetes | Bacteroidales | *Muribaculum intestinale* | 1.403 | 0 | 0.0009 | 0.0015 | 9.614 | 6.817 | 1.18 | 0.82 |
| Firmicutes | Clostridiales | *Lachnospiraceae bacterium* | 1.345 | 3.029 | 0 | 0.0003 | 0.069 | 0.27 | 0.295 | 0.4098 |
| Firmicutes | Lactobacillales | *Lactobacillus murinus* | 1.2571 | 0.011 | 0.0004 | 0 | 2.106 | 1.148 | 0 | 0 |
| Firmicutes | Lactobacillales | *Lactobacillus reuteri* | 1.1511 | 0.011 | 0 | 0 | 0.895 | 0.722 | 0 | 0 |
| Actinobacteria | Bifidobacteriales | *Bifidobacterium animalis* | 0.935 | 0.026 | 2.0006 | 0.0004 | 8.359 | 0.54 | 0.295 | 0 |
| Proteobacteria | Pseudomonadales | *Acinetobacter indicus* | 0.7679 | 0.04 | 0 | 0.008 | 0 | 0.003 | 0.59 | 0.8197 |
| Firmicutes | Clostridiales | *Clostridiales bacterium* | 0.6366 | 2.278 | 0 | 0 | 0.046 | 0.171 | 0.59 | 0.4098 |
| Proteobacteria | Pseudomonadales | *Acinetobacter towneri* | 0.543 | 0.0072 | 0.00036 | 0.0092 | 0 | 0 | 0 | 0 |
| Firmicutes | Clostridiales | *Dysosmobacter welbionis* | 0.492 | 0 | 0 | 0.0003 | 0.005 | 0.018 | 0.295 | 0 |
| Verrucomicrobia | Verrucomicrobiales | *Akkermansia muciniphila* | 0.49 | 0.194 | 0 | 0.0013 | 4.421 | 20.607 | 0 | 0 |
| Proteobacteria | Enterobacterales | *Escherichia coli* | 0.3389 | 0.128 | 3.0255 | 0.0108 | 0.022 | 0.047 | 42.48 | 3.279 |
| Firmicutes | Clostridiales | *Ruminococcus bicirculans* | 0.288 | 0.389 | 0 | 0.0003 | 0.0094 | 0 | 0 | 0 |
| Proteobacteria | Enterobacterales | *Klebsiella pneumoniae* | 0.2347 | 2.152 | 3.44 | 2.0009 | 0.118 | 3.5 | 0 | 0.4098 |
| Firmicutes | Clostridiales | *Acutalibacter muris* | 0.209 | 0.235 | 0 | 0 | 0.029 | 0.041 | 0.295 | 1.23 |
| Firmicutes | Clostridiales | *Hungatella hathewayi* | 0.2088 | 0.216 | 0 | 0 | 0.015 | 0.0093 | 0 | 0.41 |
| Firmicutes | Clostridiales | *Clostridium scindens* | 0.201 | 0.161 | 1.5 | 0 | 0.045 | 0.221 | 0 | 0.8197 |
| Firmicutes | Clostridiales | *Anaerobutyricum hallii* | 0.1838 | 0.876 | 0 | 0 | 0.033 | 0.037 | 0.29 | 0 |
| Firmicutes | Clostridiales | *Enterocloster clostridioformis* | 0.1438 | 0.446 | 0 | 0 | 0.0108 | 0.0064 | 0 | 2.049 |
| Firmicutes | Clostridiales | *Clostridium botulinum* | 0.133 | 0.353 | 4.194 | 2.5557 | 0.007 | 0.017 | 1.18 | 0.82 |
| Fibrobacteres | Fibrobacterales | *Fibrobacter succinogenes* | 0.1057 | 0.183 | 0.00036 | 0 | 0.0039 | 0.0039 | 0.59 | 0.82 |
| Bacteroidetes | Flavobacteriales | *Elizabethkingia anophelis* | 0.0721 | 0.191 | 0 | 0.0004 | 0.0037 | 0.0026 | 0 | 0.82 |
| Firmicutes | Clostridiales | *Intestinibaculum porci* | 0.0664 | 0.058 | 0 | 0 | 0.0024 | 0.0073 | 0 | 0.82 |
| Planctomycetes | Brocadiales | *Planctomycetes bacterium* | 0.0626 | 0.244 | 0 | 0.0003 | 0.0113 | 0.0113 | 0.295 | 0 |
| Firmicutes | Bacillales | *Staphylococcus nepalensis* | 0.0596 | 0 | 0 | 0 | 0.407 | 1.678 | 0 | 0.8197 |
| Proteobacteria | Pseudomonadales | *Acinetobacter baumannii* | 0.056 | 0.044 | 4.00 | 11.003 | 0.0017 | 0.0067 | 0.29 | 1.639 |
| Firmicutes | Veillonellales | *Megasphaera elsdenii* | 0.054 | 0.116 | 0 | 0 | 0.0009 | 0.0021 | 0.29 | 0 |
| Proteobacteria | Pseudomonadales | *Ralstonia pickettii* | 0.0491 | 0.007 | 0.0033 | 0.2153 | 0 | 0 | 4.13 | 9.836 |
| Proteobacteria | Rhizobiales | *Agrobacterium tumefaciens* | 0.04 | 0.059 | 0 | 0.0001 | 0.0018 | 0.0026 | 1.18 | 0 |
| Proteobacteria | Pseudomonadales | *Acinetobacter johnsonii* | 0.03 | 0.0267 | 3.5 | 0.0005 | 0 | 0.0005 | 2.37 | 8.852 |
| Proteobacteria | Burkholderiales | *Polynucleobacter necessarius* | 0.023 | 0.0306 | 0 | 0 | 0.0001 | 0.001 | 0.59 | 0 |
| Firmicutes | Bacillales | *Staphylococcus aureus* | 0.0204 | 0.021 | 4.7 | 1.6003 | 0.0144 | 0.0678 | 9.7 | 9.53 |
| Proteobacteria | Pseudomonadales | *Acinetobacter haemolyticus* | 0.019 | 0.0323 | 0 | 0.0021 | 0.0004 | 0 | 0.295 | 2.869 |
| Proteobacteria | Pseudomonadales | *Acinetobacter pittii* | 0.018 | 0.0156 | 0.0168 | 0 | 0 | 0 | 0 | 2.459 |
| Firmicutes | Lactobacillales | *Streptococcus mitis* | 0.017 | 0.0184 | 0 | 0 | 0.0007 | 0.0011 | 0.59 | 0 |
| Proteobacteria | Rhizobiales | *Sinorhizobium meliloti* | 0.014 | 0.0006 | 0.0004 | 0 | 0.0011 | 0.0004 | 0.59 | 0 |
| Proteobacteria | Vibrionales | *Vibrio alginolyticus* | 0.014 | 0.012 | 0.0008 | 0 | 0 | 0.0005 | 0 | 1.639 |
| Proteobacteria | Rhizobiales | *Ochrobactrum anthropi* | 0.013 | 0.0217 | 0 | 0 | 0.0023 | 0.002 | 0 | 0.82 |
| Proteobacteria | Pseudomonadales | *Acinetobacter cumulans* | 0.012 | 0.007 | 0 | 0.0005 | 0 | 0 | 0 | 0.41 |
| Actinobacteria | Propionibacteriales | *Cutibacterium acnes* | 0.011 | 14.034 | 0.001 | 0.0093 | 0.0005 | 0.002 | 2.06 | 0.82 |
| Proteobacteria | Pseudomonadales | *Acinetobacter junii* | 0.011 | 0.006 | 0.0003 | 0 | 0 | 0.0003 | 2.36 | 4.508 |
| Proteobacteria | Rhodobacterales | *Paracoccus yeei* | 0.01 | 0.011 | 0.0026 | 0.0017 | 0.0002 | 0.0007 | 0.59 | 0 |
| Firmicutes | Bacillales | *Staphylococcus cohnii* | 0.0092 | 0.006 | 0.0004 | 0.0008 | 0.004 | 0.234 | 0 | 0 |
| Proteobacteria | Burkholderiales | *Comamonas testosteroni* | 0.009 | 0 | 0.0003 | 0 | 0.0008 | 0.002 | 0 | 1.639 |
| Proteobacteria | Burkholderiales | *Cupriavidus metallidurans* | 0.008 | 0 | 0.0013 | 0.0377 | 0.0005 | 0.0001 | 1.18 | 2.459 |
| Proteobacteria | Burkholderiales | *Ralstonia mannitolilytica* | 0.008 | 0.0028 | 0 | 0 | 0.0002 | 0 | 0 | 1.639 |
| Proteobacteria | Campylobacterales | *Helicobacter cinaedi* | 0.0053 | 0.005 | 0 | 0.0003 | 0.0204 | 0.016 | 0.295 | 7.049 |
| Proteobacteria | Burkholderiales | *Massilia oculi* | 0.0009 | 0 | 0.0131 | 0.3521 | 0.0001 | 0 | 5.9 | 5.738 |
| Actinobacteria | Pseudonocardiales | *Actinoalloteichus sp.* | 0.0005 | 0.004 | 11.6319 | 5.249 | 0 | 0 | 0.59 | 0 |
| Proteobacteria | Enterobacterales | *Pantoea dispersa* | 0 | 0 | 24.29 | 13.861 | 0 | 0 | 0 | 0 |
| Proteobacteria | Terebellida | *Alvinella pompejana* | 0 | 0 | 0 | 0 | 0.001 | 7.827 | 0 | 0 |
| Firmicutes | Clostridiales | *Flavonifractor plautii* | 0 | 0 | 0 | 0 | 0.023 | 0.192 | 0.295 | 1.23 |
| Firmicutes | Clostridiales | Faecalibacterium prausnitzii | 0 | 0 | 0 | 0.0003 | 0.074 | 0.047 | 0 | 1.639 |
| Bacteroidetes | Bacteroidales | Bacteroides fragilis | 0 | 0 | 0.00018 | 0 | 0 | 0.193 | 0.295 | 0.82 |
| Proteobacteria | Campylobacterales | Helicobacter bilis | 0 | 0 | 0 | 0 | 0.0148 | 0.0034 | 0 | 2.869 |
| Proteobacteria | Rhodocyclales | Dechlorosoma suillum | 0 | 0.021 | 0.003 | 0.0651 | 0.0002 | 0.0001 | 0.88 | 0 |
| Bacteroidetes | Bacteroidales | Prevotella melaninogenica | 0 | 0 | 0.0002 | 10.001 | 0.0037 | 0.0113 | 0.59 | 0.41 |
| Actinobacteria | Micrococcales | Curtobacterium flaccumfaciens | 0 | 0 | 0.0017 | 0 | 0.0009 | 0.0026 | 3.835 | 2.049 |

| **Table S4:** Changes in metabolic functional profile in the study metagenomes | | | | | | | | |
| --- | --- | --- | --- | --- | --- | --- | --- | --- |
| **KO Orthologues** | | | | | | | | |
|  | **MCMF** | **HMF** | **MCMMT** | **HMMT** | **CCMF** | **HCF** | **CCMM** | **HCM** |
| ABC transporters | 57.92 | 55.52 | 80.25 | 60.75 | 59.02 | 62.25 | 75.21 | 75.55 |
| Phosphotransferase system | 17.88 | 13.17 | 1.23 | 0.00 | 6.59 | 2.70 | 5.79 | 2.06 |
| Bacterial secretion system | 24.20 | 31.31 | 18.52 | 39.25 | 34.39 | 35.05 | 19.01 | 22.39 |
| malE; maltose transport system substrate-binding protein | 36.42 | 10.76 | 0.00 | 0.34 | 18.23 | 11.77 | 0.91 | 1.45 |
| mcp; methyl-accepting chemotaxis protein | 11.48 | 18.45 | 14.18 | 20.34 | 14.38 | 22.18 | 20.67 | 34.97 |
| motB; chemotaxis protein | 9.73 | 6.19 | 23.76 | 0.00 | 4.47 | 3.42 | 8.03 | 4.84 |
| cheA; two-component system_sensor kinase | 7.57 | 12.01 | 0.00 | 7.59 | 11.45 | 14.87 | 16.87 | 8.82 |
| cheY; two-component system_response regulator | 4.93 | 9.12 | 2.13 | 11.03 | 5.34 | 5.12 | 7.18 | 4.26 |
| rbsB; ribose transport system substrate-binding protein | 4.69 | 4.04 | 0.00 | 18.62 | 4.82 | 3.87 | 1.34 | 2.75 |
| cheB; two-component system_response regulator | 4.01 | 6.64 | 6.03 | 2.41 | 6.71 | 7.82 | 3.00 | 4.77 |
| fliG; flagellar motor switch protein | 3.80 | 6.39 | 0.00 | 0.00 | 7.29 | 9.30 | 2.14 | 3.76 |
| cheV; two-component system_response regulator | 3.64 | 4.94 | 11.35 | 12.76 | 2.24 | 4.08 | 0.64 | 1.37 |
| FliN/FliY; flagellar motor switch protein | 2.59 | 3.48 | 0.00 | 5.52 | 3.87 | 5.12 | 0.91 | 1.88 |
| fliM; flagellar motor switch protein | 2.22 | 3.70 | 0.00 | 0.00 | 4.08 | 5.94 | 1.50 | 2.46 |
| cheR; chemotaxis protein methyltransferase | 1.87 | 3.22 | 0.00 | 0.00 | 2.77 | 3.68 | 1.98 | 3.61 |
| motA; chemotaxis protein | 1.87 | 2.88 | 0.00 | 0.69 | 2.73 | 3.46 | 11.78 | 3.97 |
| mglB; methyl-galactoside transport system substrate-binding protein | 1.47 | 1.58 | 0.00 | 0.00 | 4.50 | 4.14 | 8.52 | 0.07 |
| cheW; purine-binding chemotaxis protein | 1.28 | 2.12 | 0.00 | 1.72 | 1.90 | 2.21 | 1.02 | 1.45 |
| cheD; chemotaxis protein | 1.16 | 1.66 | 0.00 | 0.00 | 2.11 | 1.80 | 0.75 | 1.37 |
| cheC; chemotaxis protein | 0.67 | 1.73 | 15.25 | 6.21 | 1.64 | 1.77 | 0.43 | 0.00 |
| cheBR; two-component system_CheB/CheR fusion protein | 0.15 | 0.16 | 0.00 | 0.00 | 0.26 | 0.36 | 2.04 | 2.82 |
| cheZ; chemotaxis protein | 0.12 | 0.35 | 4.26 | 6.21 | 0.07 | 0.00 | 0.96 | 1.45 |
| cheX; chemotaxis protein | 0.08 | 0.21 | 0.00 | 0.00 | 0.57 | 0.82 | 0.00 | 0.00 |
| dppA; dipeptide transport system substrate-binding protein | 0.08 | 0.07 | 12.77 | 11.03 | 0.16 | 0.08 | 0.64 | 0.94 |
| aer; aerotaxis receptor | 0.06 | 0.17 | 3.90 | 10.69 | 0.18 | 0.11 | 3.80 | 5.56 |
| tsr; methyl-accepting chemotaxis protein I | 0.05 | 0.11 | 0.00 | 0.00 | 0.12 | 0.04 | 1.34 | 6.14 |
| tar; methyl-accepting chemotaxis protein II | 0.04 | 0.03 | 6.38 | 0.00 | 0.06 | 0.02 | 3.54 | 1.23 |
| trg; methyl-accepting chemotaxis protein III | 0.01 | 0.01 | 0.00 | 4.83 | 0.02 | 0.01 | 0.00 | 0.00 |
| tap; methyl-accepting chemotaxis protein IV | 0.00 | 0.01 | 0.00 | 0.00 | 0.02 | 0.00 | 0.00 | 0.07 |
| Bacterial chemotaxis | 67.18 | 59.16 | 16.05 | 9.80 | 58.86 | 37.56 | 56.51 | 26.77 |
| Flagellar assembly | 31.78 | 40.32 | 11.11 | 46.08 | 40.66 | 42.29 | 25.98 | 41.53 |
| Regulation of actin cytoskeleton | 1.04 | 0.53 | 72.84 | 44.12 | 0.48 | 0.15 | 47.24 | 1.96 |
| ACLY; ATP citrate (pro-S)-lyase | 0.016 | 0.016 | 2.206 | 1.044 | 0.010 | 0.000 | 18.415 | 4.134 |
| CS, gltA; citrate synthase | 9.138 | 9.688 | 0.249 | 15.608 | 7.355 | 7.476 | 0.311 | 6.489 |
| fumA/fumB; fumarate hydratase | 12.144 | 13.068 | 16.123 | 0.544 | 12.420 | 12.700 | 4.416 | 3.768 |
| fumA; fumarate hydratase subunit alpha | 1.188 | 2.314 | 9.692 | 1.543 | 3.521 | 4.826 | 4.982 | 19.257 |
| fumB; fumarate hydratase subunit beta | 0.714 | 1.266 | 11.836 | 2.359 | 2.357 | 3.025 | 1.387 | 11.983 |
| pycA; pyruvate carboxylase subunit A | 0.143 | 0.317 | 14.818 | 12.114 | 0.153 | 0.193 | 0.000 | 0.052 |
| pycB; pyruvate carboxylase subunit B | 1.965 | 2.479 | 0.000 | 0.590 | 9.407 | 7.618 | 0.000 | 0.052 |
| IDH3; isocitrate dehydrogenase | 2.269 | 1.970 | 0.000 | 0.000 | 2.818 | 3.208 | 15.202 | 0.262 |
| LSC1; succinyl-CoA synthetase alpha subunit | 0.059 | 0.109 | 11.836 | 2.541 | 0.020 | 0.007 | 0.311 | 19.257 |
| LSC2; succinyl-CoA synthetase beta subunit | 0.126 | 0.222 | 0.031 | 1.633 | 0.012 | 0.000 | 4.416 | 11.983 |
| MDH1; malate dehydrogenase | 0.003 | 0.028 | 0.000 | 3.040 | 0.003 | 0.000 | 3.241 | 0.000 |
| MDH2; malate dehydrogenase | 0.070 | 0.020 | 1.709 | 0.000 | 0.018 | 0.000 | 0.014 | 0.000 |
| PC, pyc; pyruvate carboxylase | 2.772 | 3.721 | 2.858 | 0.136 | 1.682 | 1.594 | 15.202 | 1.936 |
| acnB; aconitate hydratase 2 | 0.062 | 0.250 | 6.058 | 4.265 | 0.291 | 0.073 | 0.382 | 2.930 |
| korA; 2-oxoglutarate ferredoxin oxidoreductase subunit alpha | 21.077 | 18.884 | 0.000 | 0.000 | 23.107 | 23.361 | 5.209 | 1.256 |
| korB; 2-oxoglutarate ferredoxin oxidoreductase subunit beta | 11.917 | 11.081 | 0.000 | 0.091 | 16.229 | 16.197 | 3.241 | 0.576 |
| korD; 2-oxoglutarate ferredoxin oxidoreductase subunit delta | 0.679 | 0.735 | 3.821 | 11.207 | 0.916 | 0.863 | 15.202 | 0.000 |
| korG; 2-oxoglutarate ferredoxin oxidoreductase subunit gamma | 4.068 | 3.545 | 3.448 | 7.396 | 3.794 | 3.943 | 0.311 | 0.105 |
| mdh; malate dehydrogenase | 13.407 | 12.614 | 4.442 | 11.207 | 6.914 | 6.735 | 4.416 | 5.338 |
| sdhB; succinate dehydrogenase iron-sulfur subunit | 7.750 | 6.662 | 3.448 | 8.485 | 5.780 | 6.002 | 3.241 | 2.250 |
| sdhC; succinate dehydrogenase cytochrome b556 subunit | 2.994 | 3.658 | 3.697 | 7.895 | 1.842 | 1.430 | 0.014 | 1.256 |
| sdhD; succinate dehydrogenase membrane anchor subunit | 0.008 | 0.026 | 3.635 | 8.303 | 0.031 | 0.029 | 0.028 | 1.099 |
| sucC; succinyl-CoA synthetase beta subunit | 4.354 | 4.179 | 0.000 | 0.000 | 0.746 | 0.415 | 0.057 | 2.826 |
| sucD; succinyl-CoA synthetase alpha subunit | 3.077 | 3.149 | 0.093 | 0.000 | 0.574 | 0.305 | 0.000 | 3.192 |
| Cell communication | 38.36 | 17.33 | 43.15 | 14.29 | 32.24 | 21.19 | 41.36 | 10.01 |
| Cell growth and death | 55.50 | 51.35 | 22.72 | 22.89 | 58.49 | 56.11 | 37.38 | 28.48 |
| Cell motility | 9.90 | 15.95 | 6.89 | 18.34 | 10.20 | 14.33 | 7.34 | 42.69 |
| Transport and catabolism | 34.24 | 32.37 | 27.23 | 27.47 | 31.08 | 29.37 | 13.92 | 18.83 |
| ADA; adenosine deaminase | 32.11 | 4.42 | 15.89 | 3.78 | 13.1 | 5.77 | 19.23 | 5.7 |
| RAG1/RAG2; recombination-activating proteins | 10.14 | 0.71 | 34.21 | 21.4 | 0 | 0 | 11.44 | 2.1 |
| Two-component system | 35.668 | 21.849 | 17.821 | 9.265 | 29.814 | 20.819 | 27.663 | 8.974 |
| MAPK signaling pathway | 1.176 | 1.142 | 14.898 | 16.773 | 0.807 | 0.552 | 15.979 | 2.341 |
| ErbB signaling pathway | 0.129 | 0.084 | 2.793 | 2.875 | 0.040 | 0.002 | 1.375 | 0.285 |
| Calcium signaling pathway | 0.096 | 0.135 | 16.574 | 15.655 | 0.127 | 0.096 | 13.402 | 1.527 |
| NF-kappa B signaling pathway | 0.008 | 0.005 | 4.283 | 2.716 | 0.005 | 0.001 | 1.375 | 0.509 |
| HIF-1 signaling pathway | 20.095 | 21.161 | 17.132 | 14.217 | 19.801 | 18.832 | 4.811 | 8.243 |
| Phosphatidylinositol signaling system | 5.370 | 4.661 | 5.400 | 5.591 | 3.451 | 2.907 | 8.076 | 2.280 |
| mTOR signaling pathway | 0.079 | 0.048 | 1.862 | 0.160 | 0.025 | 0.004 | 0.687 | 0.000 |
| PI3K-Akt signaling pathway | 7.294 | 10.845 | 13.966 | 16.134 | 15.888 | 16.774 | 9.278 | 4.173 |
| Wnt signaling pathway | 0.023 | 0.032 | 8.194 | 8.946 | 0.020 | 0.007 | 9.107 | 0.794 |
| TGF-beta signaling pathway | 0.000 | 0.000 | 2.793 | 0.958 | 0.000 | 0.000 | 1.718 | 0.102 |
| Jak-STAT signaling pathway | 0.010 | 0.010 | 2.235 | 3.355 | 0.008 | 0.004 | 3.780 | 0.509 |
| **SEED Subsystems** | | | | | | | | |
| Dehydrogenase_complexes | 1.57 | 2.54 | 2.38 | 6.93 | 0.94 | 2.81 | 6.06 | 10.80 |
| Dihydroxyacetone_kinases | 0.37 | 0.49 | 0.00 | 0.00 | 0.21 | 0.25 | 2.02 | 0.91 |
| Entner-Doudoroff_Pathway | 10.09 | 8.48 | 8.33 | 7.92 | 8.40 | 8.39 | 21.21 | 7.60 |
| Ethylmalonyl-CoA_pathway_of_C2_assimilation | 0.00 | 0.01 | 0.00 | 0.00 | 0.04 | 0.09 | 0.00 | 0.58 |
| Glycolysis_and_Gluconeogenesis | 48.82 | 39.18 | 61.90 | 50.50 | 40.22 | 29.26 | 37.17 | 25.16 |
| HPr_kinase_and_hprK_operon_in_Gram-positive_organisms | 1.78 | 3.17 | 0.00 | 1.98 | 1.60 | 2.46 | 0.00 | 1.91 |
| Methylglyoxal_Metabolism | 1.07 | 1.29 | 3.57 | 8.91 | 1.45 | 1.54 | 6.06 | 5.14 |
| Particulate_methane_monooxygenase_(pMMO) | 0.00 | 0.00 | 0.00 | 0.00 | 0.00 | 0.00 | 0.00 | 0.00 |
| Pentose_phosphate_pathway | 9.88 | 7.46 | 4.76 | 7.92 | 8.63 | 7.88 | 3.03 | 7.41 |
| Peripheral_Glucose_Catabolism_Pathways | 1.14 | 1.02 | 0.00 | 0.00 | 1.17 | 1.37 | 0.00 | 0.58 |
| Pyruvate_metabolism | 25.35 | 30.40 | 11.90 | 10.89 | 31.36 | 32.95 | 34.34 | 27.13 |
| Soluble_methane_monooxygenase_(sMMO) | 0.00 | 0.00 | 0.00 | 0.00 | 0.00 | 0.01 | 0.00 | 0.06 |
| TCA_Cycle | 6.92 | 11.98 | 7.14 | 14.95 | 4.98 | 7.99 | 10.10 | 15.71 |
| Methanogenesis | 29.16 | 17.20 | 22.14 | 14.09 | 23.52 | 11.77 | 25.78 | 12.88 |
| One-carbon_metabolism | 14.68 | 5.50 | 18.75 | 9.09 | 9.01 | 6.50 | 13.03 | 4.83 |
| Serine-glyoxylate_cycle | 12.65 | 23.57 | 15.25 | 20.82 | 9.82 | 12.16 | 16.97 | 22.00 |
| Heat shock | 34.8 | 23.4 | 23.9 | 13.0 | 41.8 | 11.5 | 22.4 | 14.5 |
| Oxidative stress | 39.9 | 24.0 | 74.5 | 42.9 | 35.8 | 17.5 | 43.3 | 20.7 |
| Glutathione:_Non-redox_reactions | 15.05 | 21.68 | 12.20 | 20.69 | 23.62 | 42.86 | 22.73 | 35.42 |
| Reactive_Oxygen_Species | 42.05 | 35.24 | 43.90 | 31.03 | 44.32 | 22.23 | 29.09 | 23.99 |
| Rubrerythrin | 39.06 | 23.14 | 41.05 | 29.31 | 42.65 | 36.67 | 22.73 | 9.03 |
| Ribosome-related cluster | 23.18 | 11.17 | 18.92 | 7.19 | 12.89 | 5.64 | 21.38 | 9.56 |
| GTP or GMP signaling pathways | 9.88 | 5.46 | 11.96 | 7.14 | 13.37 | 6.46 | 15.41 | 3.82 |
| Proteolytic pathway | 23.40 | 5.22 | 34.00 | 21.51 | 27.14 | 10.02 | 46.25 | 10.45 |
| Quorum sensing and biofilm formation | 16.33 | 19.10 | 3.95 | 7.56 | 21.41 | 28.83 | 8.33 | 17.91 |
| Regulation of virulence | 61.48 | 28.68 | 31.32 | 11.20 | 63.03 | 26.65 | 43.28 | 14.58 |
| Protein_YjgK_cluster_linked_to_biofilm_formation | 11.08 | 32.14 | 13.65 | 21.48 | 7.16 | 16.06 | 10.00 | 22.78 |
| Quorum_Sensing:_Autoinducer-2_Synthesis | 38.38 | 46.81 | 9.82 | 18.52 | 5.92 | 9.63 | 1.59 | 5.78 |
| Ribosome-related cluster | 23.18 | 11.17 | 18.92 | 7.19 | 12.89 | 5.64 | 21.38 | 9.56 |
| GTP or GMP signaling pathways | 9.88 | 5.46 | 11.96 | 7.14 | 13.37 | 6.46 | 15.41 | 3.82 |
| Proteolytic pathway | 23.40 | 5.22 | 34.00 | 21.51 | 27.14 | 10.02 | 46.25 | 10.45 |
| Quorum sensing and biofilm formation | 16.33 | 19.10 | 3.95 | 7.56 | 21.41 | 28.83 | 8.33 | 17.91 |

Table S5: Archaeal genera detected (with their relative abundances) in different metagenomes.

| **Archaeal genera** | **Relative abundances (%)** | | | | | | | |
| --- | --- | --- | --- | --- | --- | --- | --- | --- |
|  | **CCMF** | **HCF** | **CCMM** | **HCM** | **MCMF** | **HCF** | **MCMMT** | **HMMT** |
| *Acidilobus* | 13.33 | 14.46 | 3.39 | 4.48 | 23.74 | 18.25 | 1.89 | 5.01 |
| *Aciduliprofundum* | 13.33 | 14.65 | 10.17 | 6.72 | 14.47 | 15.02 | 1.89 | 14.79 |
| *Aeropyrum* | 11.12 | 10.90 | 0.00 | 0.75 | 6.93 | 7.70 | 0.94 | 1.75 |
| *Archaeoglobus* | 6.48 | 6.25 | 0.00 | 0.75 | 8.34 | 8.61 | 0.00 | 11.03 |
| *Caldivirga* | 3.51 | 3.34 | 0.85 | 0.75 | 5.12 | 4.85 | 0.94 | 2.01 |
| *Candidatus* | 2.82 | 2.72 | 0.85 | 0.75 | 3.04 | 3.51 | 0.00 | 1.75 |
| *Cenarchaeum* | 3.72 | 3.33 | 1.69 | 0.75 | 2.65 | 2.77 | 0.00 | 1.75 |
| *Desulfurococcus* | 3.31 | 3.18 | 1.69 | 0.75 | 2.54 | 2.65 | 0.00 | 1.50 |
| *Ferroglobus* | 3.13 | 3.42 | 0.85 | 0.75 | 2.51 | 2.67 | 0.00 | 0.75 |
| *Ferroplasma* | 3.81 | 3.33 | 0.85 | 0.00 | 2.26 | 2.61 | 0.00 | 2.01 |
| *Halalkalicoccus* | 2.44 | 2.50 | 0.00 | 0.00 | 2.26 | 2.57 | 0.00 | 3.01 |
| *Haloarcula* | 2.90 | 2.57 | 0.85 | 0.00 | 2.01 | 2.25 | 0.00 | 1.50 |
| *Halobacterium* | 2.77 | 2.27 | 0.85 | 0.75 | 1.79 | 1.91 | 0.94 | 4.26 |
| *Haloferax* | 1.55 | 1.65 | 2.54 | 0.00 | 1.98 | 2.31 | 0.00 | 1.25 |
| *Halogeometricum* | 2.33 | 2.30 | 2.54 | 0.75 | 1.67 | 1.86 | 0.00 | 2.76 |
| *Halomicrobium* | 1.95 | 2.44 | 0.00 | 0.00 | 1.63 | 1.73 | 0.00 | 2.01 |
| *Haloquadratum* | 1.47 | 1.88 | 0.00 | 0.00 | 1.40 | 1.47 | 0.94 | 2.51 |
| *Halorhabdus* | 0.87 | 0.79 | 0.00 | 0.75 | 1.09 | 1.28 | 0.00 | 0.50 |
| *Halorubrum* | 1.36 | 1.30 | 0.00 | 0.75 | 0.88 | 0.95 | 0.00 | 1.25 |
| *Haloterrigena* | 1.05 | 0.86 | 0.00 | 0.00 | 0.90 | 1.06 | 0.00 | 1.25 |
| *Hyperthermus* | 1.07 | 1.11 | 0.00 | 0.00 | 0.91 | 0.92 | 0.00 | 1.50 |
| *Ignicoccus* | 1.07 | 1.02 | 0.00 | 0.00 | 0.83 | 1.03 | 0.00 | 2.01 |
| *Ignisphaera* | 0.84 | 0.83 | 0.00 | 0.00 | 0.84 | 0.95 | 0.00 | 0.25 |
| *Metallosphaera* | 0.89 | 0.97 | 5.08 | 0.00 | 0.57 | 0.68 | 0.94 | 0.50 |
| *Methanobrevibacter* | 1.06 | 0.90 | 0.00 | 0.00 | 0.56 | 0.57 | 1.89 | 1.50 |
| *Methanocaldococcus* | 0.40 | 0.36 | 1.69 | 0.75 | 0.77 | 0.78 | 0.00 | 0.00 |
| *Methanocella* | 0.64 | 0.72 | 0.85 | 0.00 | 0.55 | 0.62 | 0.00 | 0.50 |
| *Methanococcoides* | 0.57 | 0.64 | 0.00 | 0.00 | 0.52 | 0.60 | 0.00 | 0.25 |
| *Methanococcus* | 0.65 | 0.59 | 0.00 | 1.49 | 0.51 | 0.54 | 0.00 | 0.75 |
| *Methanocorpusculum* | 0.61 | 0.49 | 16.95 | 14.18 | 0.42 | 0.42 | 0.94 | 0.75 |
| *Methanoculleus* | 0.46 | 0.41 | 5.08 | 4.48 | 0.44 | 0.51 | 1.89 | 2.51 |
| *Methanohalobium* | 0.62 | 0.88 | 0.00 | 2.24 | 0.32 | 0.34 | 0.00 | 1.50 |
| *Methanohalophilus* | 0.54 | 0.54 | 0.00 | 0.00 | 0.36 | 0.38 | 0.00 | 0.50 |
| *Methanoplanus* | 0.71 | 0.43 | 2.54 | 2.99 | 0.29 | 0.32 | 22.64 | 6.02 |
| *Methanopyrus* | 0.39 | 0.39 | 0.00 | 0.75 | 0.36 | 0.43 | 0.00 | 0.25 |
| *Methanoregula* | 0.53 | 0.41 | 0.00 | 0.00 | 0.32 | 0.30 | 0.00 | 1.50 |
| *Methanosaeta* | 0.41 | 0.42 | 2.54 | 2.99 | 0.32 | 0.32 | 46.23 | 9.27 |
| *Methanosarcina* | 0.40 | 0.31 | 1.69 | 2.99 | 0.33 | 0.39 | 3.77 | 2.01 |
| *Methanosphaera* | 0.25 | 0.19 | 0.00 | 0.00 | 0.38 | 0.42 | 0.00 | 0.50 |
| *Methanosphaerula* | 0.37 | 0.36 | 33.05 | 42.54 | 0.25 | 0.26 | 11.32 | 1.25 |
| *Methanospirillum* | 0.33 | 0.44 | 0.85 | 0.00 | 0.28 | 0.26 | 0.00 | 0.75 |
| *Methanothermobacter* | 0.28 | 0.37 | 0.00 | 0.00 | 0.30 | 0.27 | 0.00 | 0.25 |
| *Methanothermococcus* | 0.45 | 0.30 | 0.00 | 0.00 | 0.23 | 0.29 | 0.00 | 0.00 |
| *Methanothermus* | 0.25 | 0.30 | 0.00 | 1.49 | 0.27 | 0.28 | 0.00 | 0.00 |
| *Nanoarchaeum* | 0.26 | 0.29 | 0.00 | 0.75 | 0.24 | 0.29 | 0.94 | 0.75 |
| *Natrialba* | 0.23 | 0.17 | 0.00 | 0.00 | 0.24 | 0.33 | 0.00 | 0.00 |
| *Natronomonas* | 0.36 | 0.29 | 0.00 | 0.00 | 0.20 | 0.21 | 0.00 | 0.00 |
| *Nitrosopumilus* | 0.34 | 0.32 | 0.00 | 0.00 | 0.21 | 0.19 | 0.00 | 0.00 |
| *Picrophilus* | 0.29 | 0.23 | 0.00 | 0.00 | 0.20 | 0.27 | 0.00 | 0.25 |
| *Pyrobaculum* | 0.34 | 0.30 | 0.00 | 0.00 | 0.17 | 0.18 | 0.00 | 0.75 |
| *Pyrococcus* | 0.32 | 0.23 | 2.54 | 2.99 | 0.17 | 0.17 | 1.89 | 0.75 |
| *Staphylothermus* | 0.38 | 0.34 | 0.00 | 0.00 | 0.13 | 0.16 | 0.00 | 0.50 |
| *Sulfolobus* | 0.26 | 0.16 | 0.00 | 0.00 | 0.12 | 0.13 | 0.00 | 0.00 |
| *Thermococcus* | 0.16 | 0.15 | 0.00 | 0.00 | 0.13 | 0.17 | 0.00 | 0.00 |
