# Supplementary material for: Induction of mastitis by cow-to-mouse fecal and milk microbiota transplantation causes microbiome dysbiosis and genomic functional perturbation in mice
Source: Anim Microbiome. 2022 Jul 6;4:43. doi: 10.1186/s42523-022-00193-w (PMC9258091; doi:10.1186/s42523-022-00193-w)
Supplement: Supplementary file 3 — Additional file 3. Taxonomic structure of microbiomes identified in different sample groups. [file 42523_2022_193_MOESM3_ESM.docx]

**Additional file 3:** Taxonomic structure of microbiomes identified in different sample groups.

| **Taxonomic ranks** | **Metagenome groups and number of taxa detected** | | | | | | | |
| --- | --- | --- | --- | --- | --- | --- | --- | --- |
|  | **CCMF** | **HCF** | **CCMM** | **HCM** | **MCMF** | **HMF** | **MCMMT** | **HMMT** |
| Bacterial species (n = 2191) | **1731** | | **618** | | **1065** | | **115** | |
|  | 1590 | 979 | 592 | 79 | 853 | 561 | 69 | 77 |
| Viral genera (n = 94) | 80 | 63 | 13 | 27 | 69 | 56 | 20 | 21 |
| Archaeal genera (n = 60) | 60 | 60 | 16 | 49 | 60 | 60 | 23 | 26 |
