# Supplementary figures and images for: Induction of mastitis by cow-to-mouse fecal and milk microbiota transplantation causes microbiome dysbiosis and genomic functional perturbation in mice
Source: Anim Microbiome. 2022 Jul 6;4:43. doi: 10.1186/s42523-022-00193-w (PMC9258091; doi:10.1186/s42523-022-00193-w)

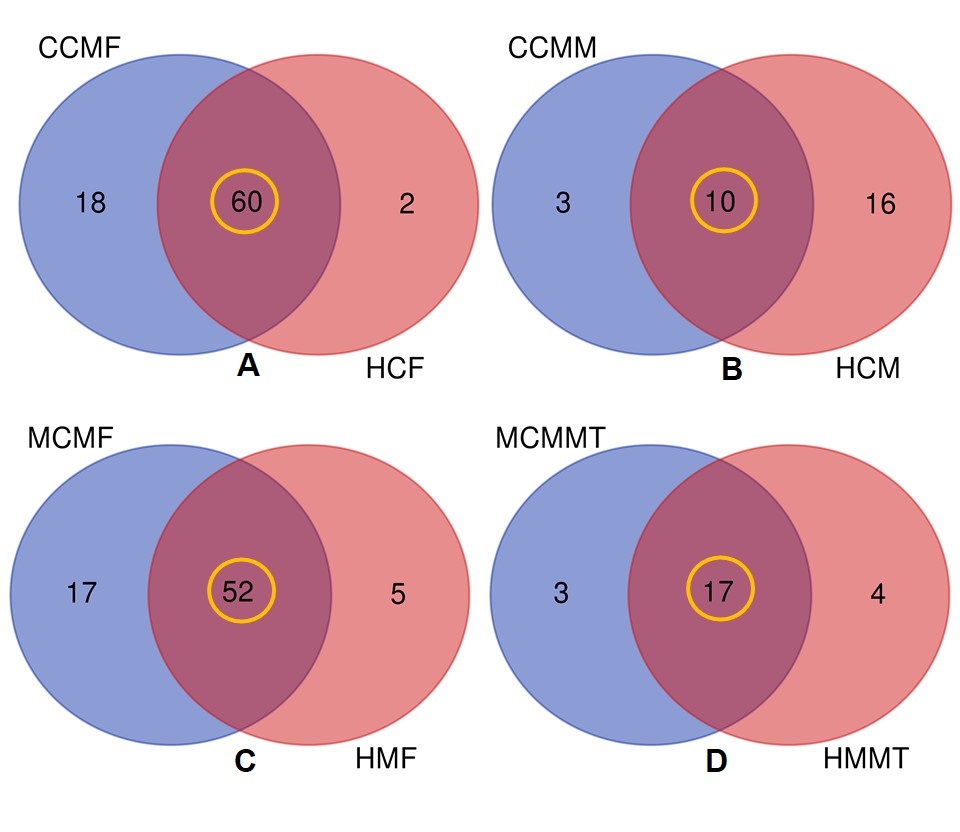

Supplement: Supplementary file 4 — Additional file 4. Taxonomic composition of viruses. [file 42523_2022_193_MOESM4_ESM.jpg]

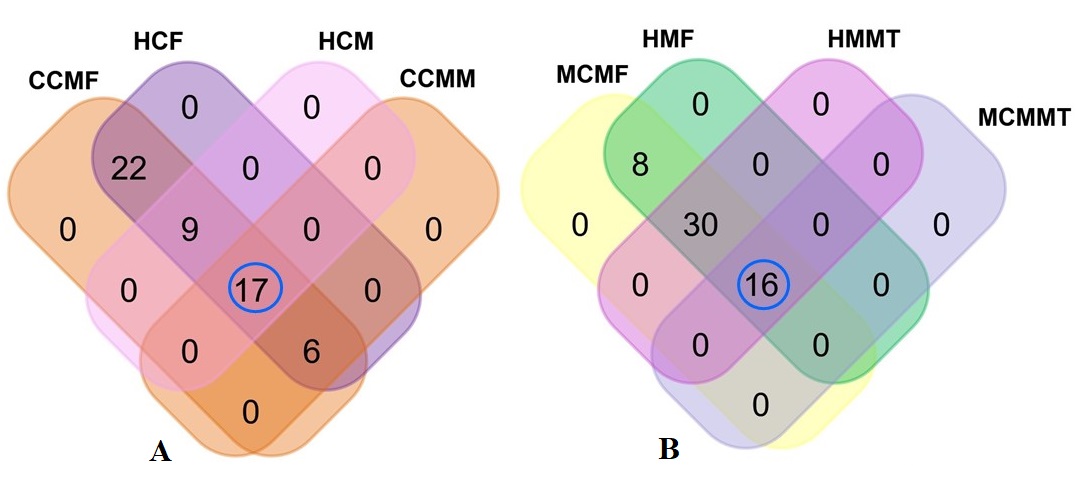

Supplement: Supplementary file 5 — Additional file 5. Taxonomic composition of archaea. [file 42523_2022_193_MOESM5_ESM.jpg]

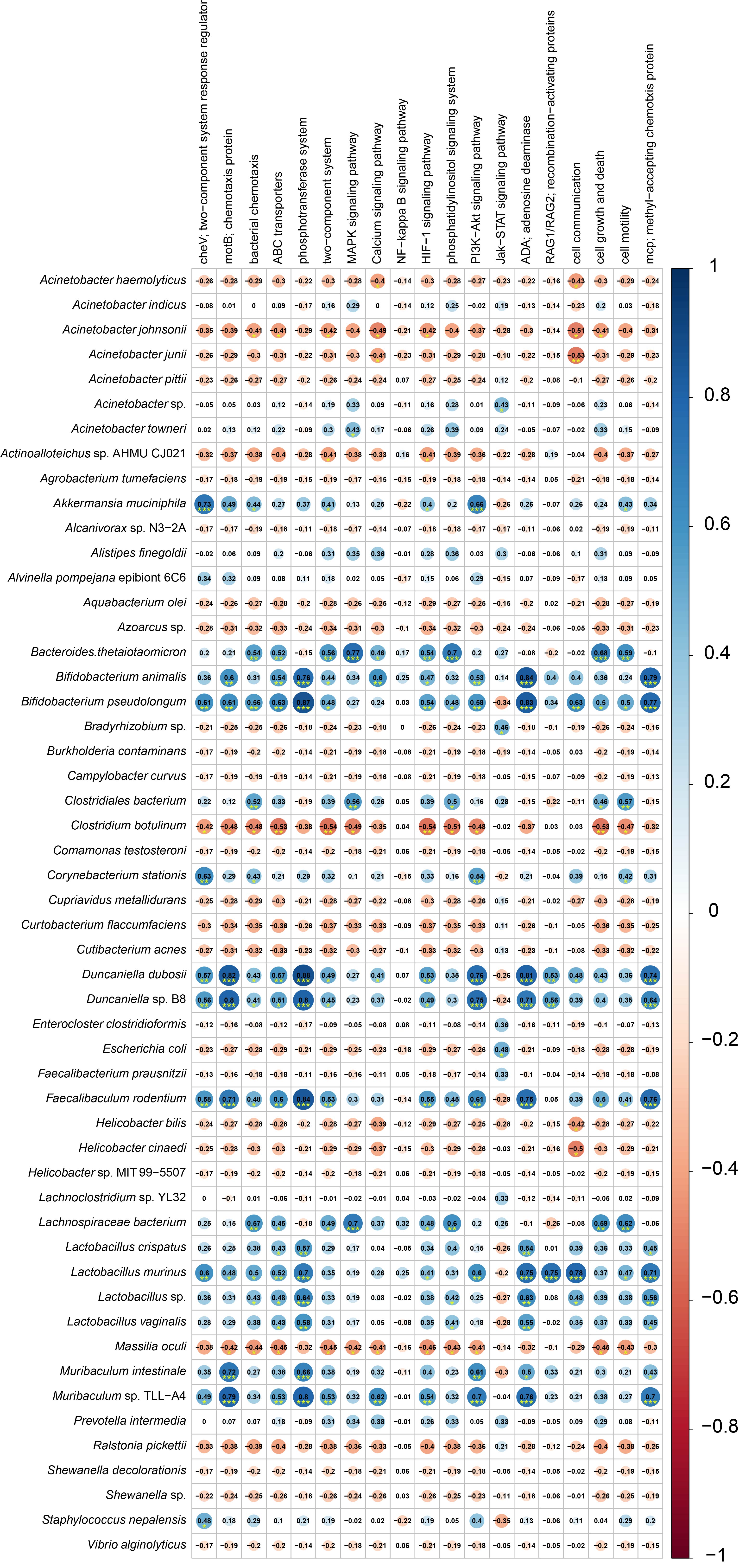

Supplement: Supplementary file 9 — Additional file 9. The correlation between predominantly abundant bacterial species and their genomic functional potentials in different KEGG orthologues (KOs) in CM and H control metagenomes of both cows and mice. [file 42523_2022_193_MOESM9_ESM.jpg]
