# Supplementary material for: Induction of mastitis by cow-to-mouse fecal and milk microbiota transplantation causes microbiome dysbiosis and genomic functional perturbation in mice
Source: Anim Microbiome. 2022 Jul 6;4:43. doi: 10.1186/s42523-022-00193-w (PMC9258091; doi:10.1186/s42523-022-00193-w)
Supplement: Supplementary file 6 — Additional file 6. Taxonomic information of top seventy bacterial species identified in different metagenomic groups. [file 42523_2022_193_MOESM6_ESM.docx]

| **Additional file 6: Taxonomic information of top seventy bacterial species identified in different metagenomic groups.** | | | | | | | | | | |
| --- | --- | --- | --- | --- | --- | --- | --- | --- | --- | --- |
| **Phyla** | **Order** | **Species** | **CCMF** | **HCF** | **CCMM** | **HCM** | **MCMF** | **HMF** | **MCMMT** | **HMMT** |
| Proteobacteria | Pseudomonadales | *Pseudomonas aeruginosa* | 21.001 | 32.7 | 2.0131 | 40.352 | 0.0001 | 3.2 | 2.9 | 8.738 |
| Firmicutes | Lactobacillales | *Lactobacillus crispatus* | 12.102 | 0.008 | 0 | 0 | 2.873 | 1.798 | 0 | 0 |
| Proteobacteria | Enterobacterales | *Klebsiella oxytoca* | 10.324 | 2.9 | 20.68 | 4.0006 | 0.946 | 1.012 | 0 | 0.41 |
| Firmicutes | Lactobacillales | *Enterococcus faecalis* | 10.02 | 30.052 | 0.00036 | 0 | 5.0068 | 2.0201 | 0.59 | 0.41 |
| Proteobacteria | Burkholderiales | *Nocardia pseudobrasiliensis* | 5.002 | 2.0022 | 8.87 | 3.046 | 0 | 0 | 0.88 | 0.82 |
| Firmicutes | Lactobacillales | *Lactobacillus vaginalis* | 4.8543 | 0.003 | 0 | 0 | 0.934 | 0.961 | 0 | 0 |
| Firmicutes | Clostridiales | *Clostridioides difficile* | 4.231 | 0.77 | 1.7 | 0.0006 | 0.027 | 0.01 | 0.295 | 0 |
| Proteobacteria | Pseudomonadales | *Ralstonia insidiosa* | 4.005 | 2.005 | 2.5001 | 0.0297 | 0 | 0 | 0.59 | 0 |
| Actinobacteria | Bifidobacteriales | *Bifidobacterium pseudolongum* | 3.08 | 0.042 | 0.0003 | 0.0004 | 2.151 | 1.649 | 0 | 0 |
| Bacteroidetes | Bacteroidales | *Muribaculum sp.* | 2.354 | 0 | 0.001 | 0.0028 | 38.301 | 26.738 | 1.77 | 0 |
| Bacteroidetes | Bacteroidales | *Duncaniella sp.* | 2.325 | 0.576 | 0 | 0.0003 | 10.168 | 7.893 | 1.77 | 0.82 |
| Bacteroidetes | Bacteroidales | *Duncaniella dubosii* | 2.008 | 0 | 0.0005 | 0.0007 | 7.141 | 4.71 | 0.885 | 0.82 |
| Firmicutes | Erysipelotrichales | *Faecalibaculum rodentium* | 1.7801 | 0.169 | 0 | 0.0003 | 1.859 | 1.288 | 0 | 0 |
| Bacteroidetes | Bacteroidales | *Muribaculum intestinale* | 1.403 | 0 | 0.0009 | 0.0015 | 9.614 | 6.817 | 1.18 | 0.82 |
| Firmicutes | Clostridiales | *Lachnospiraceae bacterium* | 1.345 | 3.029 | 0 | 0.0003 | 0.069 | 0.27 | 0.295 | 0.4098 |
| Firmicutes | Lactobacillales | *Lactobacillus murinus* | 1.2571 | 0.011 | 0.0004 | 0 | 2.106 | 1.148 | 0 | 0 |
| Firmicutes | Lactobacillales | *Lactobacillus reuteri* | 1.1511 | 0.011 | 0 | 0 | 0.895 | 0.722 | 0 | 0 |
| Actinobacteria | Bifidobacteriales | *Bifidobacterium animalis* | 0.935 | 0.026 | 2.0006 | 0.0004 | 8.359 | 0.54 | 0.295 | 0 |
| Proteobacteria | Pseudomonadales | *Acinetobacter indicus* | 0.7679 | 0.04 | 0 | 0.008 | 0 | 0.003 | 0.59 | 0.8197 |
| Firmicutes | Clostridiales | *Clostridiales bacterium* | 0.6366 | 2.278 | 0 | 0 | 0.046 | 0.171 | 0.59 | 0.4098 |
| Proteobacteria | Pseudomonadales | *Acinetobacter towneri* | 0.543 | 0.0072 | 0.00036 | 0.0092 | 0 | 0 | 0 | 0 |
| Firmicutes | Clostridiales | *Dysosmobacter welbionis* | 0.492 | 0 | 0 | 0.0003 | 0.005 | 0.018 | 0.295 | 0 |
| Verrucomicrobia | Verrucomicrobiales | *Akkermansia muciniphila* | 0.49 | 0.194 | 0 | 0.0013 | 4.421 | 20.607 | 0 | 0 |
| Proteobacteria | Enterobacterales | *Escherichia coli* | 0.3389 | 0.128 | 3.0255 | 0.0108 | 0.022 | 0.047 | 42.48 | 3.279 |
| Firmicutes | Clostridiales | *Ruminococcus bicirculans* | 0.288 | 0.389 | 0 | 0.0003 | 0.0094 | 0 | 0 | 0 |
| Proteobacteria | Enterobacterales | *Klebsiella pneumoniae* | 0.2347 | 2.152 | 3.44 | 2.0009 | 0.118 | 3.5 | 0 | 0.4098 |
| Firmicutes | Clostridiales | *Acutalibacter muris* | 0.209 | 0.235 | 0 | 0 | 0.029 | 0.041 | 0.295 | 1.23 |
| Firmicutes | Clostridiales | *Hungatella hathewayi* | 0.2088 | 0.216 | 0 | 0 | 0.015 | 0.0093 | 0 | 0.41 |
| Firmicutes | Clostridiales | *Clostridium scindens* | 0.201 | 0.161 | 1.5 | 0 | 0.045 | 0.221 | 0 | 0.8197 |
| Firmicutes | Clostridiales | *Anaerobutyricum hallii* | 0.1838 | 0.876 | 0 | 0 | 0.033 | 0.037 | 0.29 | 0 |
| Firmicutes | Clostridiales | *Enterocloster clostridioformis* | 0.1438 | 0.446 | 0 | 0 | 0.0108 | 0.0064 | 0 | 2.049 |
| Firmicutes | Clostridiales | *Clostridium botulinum* | 0.133 | 0.353 | 4.194 | 2.5557 | 0.007 | 0.017 | 1.18 | 0.82 |
| Fibrobacteres | Fibrobacterales | *Fibrobacter succinogenes* | 0.1057 | 0.183 | 0.00036 | 0 | 0.0039 | 0.0039 | 0.59 | 0.82 |
| Bacteroidetes | Flavobacteriales | *Elizabethkingia anophelis* | 0.0721 | 0.191 | 0 | 0.0004 | 0.0037 | 0.0026 | 0 | 0.82 |
| Firmicutes | Clostridiales | *Intestinibaculum porci* | 0.0664 | 0.058 | 0 | 0 | 0.0024 | 0.0073 | 0 | 0.82 |
| Planctomycetes | Brocadiales | *Planctomycetes bacterium* | 0.0626 | 0.244 | 0 | 0.0003 | 0.0113 | 0.0113 | 0.295 | 0 |
| Firmicutes | Bacillales | *Staphylococcus nepalensis* | 0.0596 | 0 | 0 | 0 | 0.407 | 1.678 | 0 | 0.8197 |
| Proteobacteria | Pseudomonadales | *Acinetobacter baumannii* | 0.056 | 0.044 | 4.00 | 11.003 | 0.0017 | 0.0067 | 0.29 | 1.639 |
| Firmicutes | Veillonellales | *Megasphaera elsdenii* | 0.054 | 0.116 | 0 | 0 | 0.0009 | 0.0021 | 0.29 | 0 |
| Proteobacteria | Pseudomonadales | *Ralstonia pickettii* | 0.0491 | 0.007 | 0.0033 | 0.2153 | 0 | 0 | 4.13 | 9.836 |
| Proteobacteria | Rhizobiales | *Agrobacterium tumefaciens* | 0.04 | 0.059 | 0 | 0.0001 | 0.0018 | 0.0026 | 1.18 | 0 |
| Proteobacteria | Pseudomonadales | *Acinetobacter johnsonii* | 0.03 | 0.0267 | 3.5 | 0.0005 | 0 | 0.0005 | 2.37 | 8.852 |
| Proteobacteria | Burkholderiales | *Polynucleobacter necessarius* | 0.023 | 0.0306 | 0 | 0 | 0.0001 | 0.001 | 0.59 | 0 |
| Firmicutes | Bacillales | *Staphylococcus aureus* | 0.0204 | 0.021 | 4.7 | 1.6003 | 0.0144 | 0.0678 | 9.7 | 9.53 |
| Proteobacteria | Pseudomonadales | *Acinetobacter haemolyticus* | 0.019 | 0.0323 | 0 | 0.0021 | 0.0004 | 0 | 0.295 | 2.869 |
| Proteobacteria | Pseudomonadales | *Acinetobacter pittii* | 0.018 | 0.0156 | 0.0168 | 0 | 0 | 0 | 0 | 2.459 |
| Firmicutes | Lactobacillales | *Streptococcus mitis* | 0.017 | 0.0184 | 0 | 0 | 0.0007 | 0.0011 | 0.59 | 0 |
| Proteobacteria | Rhizobiales | *Sinorhizobium meliloti* | 0.014 | 0.0006 | 0.0004 | 0 | 0.0011 | 0.0004 | 0.59 | 0 |
| Proteobacteria | Vibrionales | *Vibrio alginolyticus* | 0.014 | 0.012 | 0.0008 | 0 | 0 | 0.0005 | 0 | 1.639 |
| Proteobacteria | Rhizobiales | *Ochrobactrum anthropi* | 0.013 | 0.0217 | 0 | 0 | 0.0023 | 0.002 | 0 | 0.82 |
| Proteobacteria | Pseudomonadales | *Acinetobacter cumulans* | 0.012 | 0.007 | 0 | 0.0005 | 0 | 0 | 0 | 0.41 |
| Actinobacteria | Propionibacteriales | *Cutibacterium acnes* | 0.011 | 14.034 | 0.001 | 0.0093 | 0.0005 | 0.002 | 2.06 | 0.82 |
| Proteobacteria | Pseudomonadales | *Acinetobacter junii* | 0.011 | 0.006 | 0.0003 | 0 | 0 | 0.0003 | 2.36 | 4.508 |
| Proteobacteria | Rhodobacterales | *Paracoccus yeei* | 0.01 | 0.011 | 0.0026 | 0.0017 | 0.0002 | 0.0007 | 0.59 | 0 |
| Firmicutes | Bacillales | *Staphylococcus cohnii* | 0.0092 | 0.006 | 0.0004 | 0.0008 | 0.004 | 0.234 | 0 | 0 |
| Proteobacteria | Burkholderiales | *Comamonas testosteroni* | 0.009 | 0 | 0.0003 | 0 | 0.0008 | 0.002 | 0 | 1.639 |
| Proteobacteria | Burkholderiales | *Cupriavidus metallidurans* | 0.008 | 0 | 0.0013 | 0.0377 | 0.0005 | 0.0001 | 1.18 | 2.459 |
| Proteobacteria | Burkholderiales | *Ralstonia mannitolilytica* | 0.008 | 0.0028 | 0 | 0 | 0.0002 | 0 | 0 | 1.639 |
| Proteobacteria | Campylobacterales | *Helicobacter cinaedi* | 0.0053 | 0.005 | 0 | 0.0003 | 0.0204 | 0.016 | 0.295 | 7.049 |
| Proteobacteria | Burkholderiales | *Massilia oculi* | 0.0009 | 0 | 0.0131 | 0.3521 | 0.0001 | 0 | 5.9 | 5.738 |
| Actinobacteria | Pseudonocardiales | *Actinoalloteichus sp.* | 0.0005 | 0.004 | 11.6319 | 5.249 | 0 | 0 | 0.59 | 0 |
| Proteobacteria | Enterobacterales | *Pantoea dispersa* | 0 | 0 | 24.29 | 13.861 | 0 | 0 | 0 | 0 |
| Proteobacteria | Terebellida | *Alvinella pompejana* | 0 | 0 | 0 | 0 | 0.001 | 7.827 | 0 | 0 |
| Firmicutes | Clostridiales | *Flavonifractor plautii* | 0 | 0 | 0 | 0 | 0.023 | 0.192 | 0.295 | 1.23 |
| Firmicutes | Clostridiales | Faecalibacterium prausnitzii | 0 | 0 | 0 | 0.0003 | 0.074 | 0.047 | 0 | 1.639 |
| Bacteroidetes | Bacteroidales | Bacteroides fragilis | 0 | 0 | 0.00018 | 0 | 0 | 0.193 | 0.295 | 0.82 |
| Proteobacteria | Campylobacterales | Helicobacter bilis | 0 | 0 | 0 | 0 | 0.0148 | 0.0034 | 0 | 2.869 |
| Proteobacteria | Rhodocyclales | Dechlorosoma suillum | 0 | 0.021 | 0.003 | 0.0651 | 0.0002 | 0.0001 | 0.88 | 0 |
| Bacteroidetes | Bacteroidales | Prevotella melaninogenica | 0 | 0 | 0.0002 | 10.001 | 0.0037 | 0.0113 | 0.59 | 0.41 |
| Actinobacteria | Micrococcales | Curtobacterium flaccumfaciens | 0 | 0 | 0.0017 | 0 | 0.0009 | 0.0026 | 3.835 | 2.049 |
