# Supplementary material for: Induction of mastitis by cow-to-mouse fecal and milk microbiota transplantation causes microbiome dysbiosis and genomic functional perturbation in mice
Source: Anim Microbiome. 2022 Jul 6;4:43. doi: 10.1186/s42523-022-00193-w (PMC9258091; doi:10.1186/s42523-022-00193-w)
Supplement: Supplementary file 7 — Additional file 7. Archaeal genera detected (with their relative abundances) in different metagenomes. [file 42523_2022_193_MOESM7_ESM.docx]

**Additional file 7**: Archaeal genera detected (with their relative abundances) in different metagenomes.

| **Archaeal genera** | **Relative abundances (%)** | | | | | | | |
| --- | --- | --- | --- | --- | --- | --- | --- | --- |
|  | **CCMF** | **HCF** | **CCMM** | **HCM** | **MCMF** | **HCF** | **MCMMT** | **HMMT** |
| *Acidilobus* | 13.33 | 14.46 | 3.39 | 4.48 | 23.74 | 18.25 | 1.89 | 5.01 |
| *Aciduliprofundum* | 13.33 | 14.65 | 10.17 | 6.72 | 14.47 | 15.02 | 1.89 | 14.79 |
| *Aeropyrum* | 11.12 | 10.90 | 0.00 | 0.75 | 6.93 | 7.70 | 0.94 | 1.75 |
| *Archaeoglobus* | 6.48 | 6.25 | 0.00 | 0.75 | 8.34 | 8.61 | 0.00 | 11.03 |
| *Caldivirga* | 3.51 | 3.34 | 0.85 | 0.75 | 5.12 | 4.85 | 0.94 | 2.01 |
| *Candidatus* | 2.82 | 2.72 | 0.85 | 0.75 | 3.04 | 3.51 | 0.00 | 1.75 |
| *Cenarchaeum* | 3.72 | 3.33 | 1.69 | 0.75 | 2.65 | 2.77 | 0.00 | 1.75 |
| *Desulfurococcus* | 3.31 | 3.18 | 1.69 | 0.75 | 2.54 | 2.65 | 0.00 | 1.50 |
| *Ferroglobus* | 3.13 | 3.42 | 0.85 | 0.75 | 2.51 | 2.67 | 0.00 | 0.75 |
| *Ferroplasma* | 3.81 | 3.33 | 0.85 | 0.00 | 2.26 | 2.61 | 0.00 | 2.01 |
| *Halalkalicoccus* | 2.44 | 2.50 | 0.00 | 0.00 | 2.26 | 2.57 | 0.00 | 3.01 |
| *Haloarcula* | 2.90 | 2.57 | 0.85 | 0.00 | 2.01 | 2.25 | 0.00 | 1.50 |
| *Halobacterium* | 2.77 | 2.27 | 0.85 | 0.75 | 1.79 | 1.91 | 0.94 | 4.26 |
| *Haloferax* | 1.55 | 1.65 | 2.54 | 0.00 | 1.98 | 2.31 | 0.00 | 1.25 |
| *Halogeometricum* | 2.33 | 2.30 | 2.54 | 0.75 | 1.67 | 1.86 | 0.00 | 2.76 |
| *Halomicrobium* | 1.95 | 2.44 | 0.00 | 0.00 | 1.63 | 1.73 | 0.00 | 2.01 |
| *Haloquadratum* | 1.47 | 1.88 | 0.00 | 0.00 | 1.40 | 1.47 | 0.94 | 2.51 |
| *Halorhabdus* | 0.87 | 0.79 | 0.00 | 0.75 | 1.09 | 1.28 | 0.00 | 0.50 |
| *Halorubrum* | 1.36 | 1.30 | 0.00 | 0.75 | 0.88 | 0.95 | 0.00 | 1.25 |
| *Haloterrigena* | 1.05 | 0.86 | 0.00 | 0.00 | 0.90 | 1.06 | 0.00 | 1.25 |
| *Hyperthermus* | 1.07 | 1.11 | 0.00 | 0.00 | 0.91 | 0.92 | 0.00 | 1.50 |
| *Ignicoccus* | 1.07 | 1.02 | 0.00 | 0.00 | 0.83 | 1.03 | 0.00 | 2.01 |
| *Ignisphaera* | 0.84 | 0.83 | 0.00 | 0.00 | 0.84 | 0.95 | 0.00 | 0.25 |
| *Metallosphaera* | 0.89 | 0.97 | 5.08 | 0.00 | 0.57 | 0.68 | 0.94 | 0.50 |
| *Methanobrevibacter* | 1.06 | 0.90 | 0.00 | 0.00 | 0.56 | 0.57 | 1.89 | 1.50 |
| *Methanocaldococcus* | 0.40 | 0.36 | 1.69 | 0.75 | 0.77 | 0.78 | 0.00 | 0.00 |
| *Methanocella* | 0.64 | 0.72 | 0.85 | 0.00 | 0.55 | 0.62 | 0.00 | 0.50 |
| *Methanococcoides* | 0.57 | 0.64 | 0.00 | 0.00 | 0.52 | 0.60 | 0.00 | 0.25 |
| *Methanococcus* | 0.65 | 0.59 | 0.00 | 1.49 | 0.51 | 0.54 | 0.00 | 0.75 |
| *Methanocorpusculum* | 0.61 | 0.49 | 16.95 | 14.18 | 0.42 | 0.42 | 0.94 | 0.75 |
| *Methanoculleus* | 0.46 | 0.41 | 5.08 | 4.48 | 0.44 | 0.51 | 1.89 | 2.51 |
| *Methanohalobium* | 0.62 | 0.88 | 0.00 | 2.24 | 0.32 | 0.34 | 0.00 | 1.50 |
| *Methanohalophilus* | 0.54 | 0.54 | 0.00 | 0.00 | 0.36 | 0.38 | 0.00 | 0.50 |
| *Methanoplanus* | 0.71 | 0.43 | 2.54 | 2.99 | 0.29 | 0.32 | 22.64 | 6.02 |
| *Methanopyrus* | 0.39 | 0.39 | 0.00 | 0.75 | 0.36 | 0.43 | 0.00 | 0.25 |
| *Methanoregula* | 0.53 | 0.41 | 0.00 | 0.00 | 0.32 | 0.30 | 0.00 | 1.50 |
| *Methanosaeta* | 0.41 | 0.42 | 2.54 | 2.99 | 0.32 | 0.32 | 46.23 | 9.27 |
| *Methanosarcina* | 0.40 | 0.31 | 1.69 | 2.99 | 0.33 | 0.39 | 3.77 | 2.01 |
| *Methanosphaera* | 0.25 | 0.19 | 0.00 | 0.00 | 0.38 | 0.42 | 0.00 | 0.50 |
| *Methanosphaerula* | 0.37 | 0.36 | 33.05 | 42.54 | 0.25 | 0.26 | 11.32 | 1.25 |
| *Methanospirillum* | 0.33 | 0.44 | 0.85 | 0.00 | 0.28 | 0.26 | 0.00 | 0.75 |
| *Methanothermobacter* | 0.28 | 0.37 | 0.00 | 0.00 | 0.30 | 0.27 | 0.00 | 0.25 |
| *Methanothermococcus* | 0.45 | 0.30 | 0.00 | 0.00 | 0.23 | 0.29 | 0.00 | 0.00 |
| *Methanothermus* | 0.25 | 0.30 | 0.00 | 1.49 | 0.27 | 0.28 | 0.00 | 0.00 |
| *Nanoarchaeum* | 0.26 | 0.29 | 0.00 | 0.75 | 0.24 | 0.29 | 0.94 | 0.75 |
| *Natrialba* | 0.23 | 0.17 | 0.00 | 0.00 | 0.24 | 0.33 | 0.00 | 0.00 |
| *Natronomonas* | 0.36 | 0.29 | 0.00 | 0.00 | 0.20 | 0.21 | 0.00 | 0.00 |
| *Nitrosopumilus* | 0.34 | 0.32 | 0.00 | 0.00 | 0.21 | 0.19 | 0.00 | 0.00 |
| *Picrophilus* | 0.29 | 0.23 | 0.00 | 0.00 | 0.20 | 0.27 | 0.00 | 0.25 |
| *Pyrobaculum* | 0.34 | 0.30 | 0.00 | 0.00 | 0.17 | 0.18 | 0.00 | 0.75 |
| *Pyrococcus* | 0.32 | 0.23 | 2.54 | 2.99 | 0.17 | 0.17 | 1.89 | 0.75 |
| *Staphylothermus* | 0.38 | 0.34 | 0.00 | 0.00 | 0.13 | 0.16 | 0.00 | 0.50 |
| *Sulfolobus* | 0.26 | 0.16 | 0.00 | 0.00 | 0.12 | 0.13 | 0.00 | 0.00 |
| *Thermococcus* | 0.16 | 0.15 | 0.00 | 0.00 | 0.13 | 0.17 | 0.00 | 0.00 |
